# Quercetin Attenuates Trauma-Induced Heterotopic Ossification by Tuning Immune Cell Infiltration and Related Inflammatory Insult

**DOI:** 10.3389/fimmu.2021.649285

**Published:** 2021-05-20

**Authors:** Juehong Li, Ziyang Sun, Gang Luo, Shuo Wang, Haomin Cui, Zhixiao Yao, Hao Xiong, Yunwei He, Yun Qian, Cunyi Fan

**Affiliations:** ^1^ Department of Orthopaedic Surgery, Shanghai Jiao Tong University Affiliated Sixth People’s Hospital, Shanghai, China; ^2^ Youth Science and Technology Innovation Studio of Shanghai Jiao Tong University School of Medicine, Shanghai, China

**Keywords:** quercetin, heterotopic ossification, SIRT1, mast cells, macrophages

## Abstract

Heterotopic ossification (HO) is one of the most intractable disorders following musculoskeletal injury and is characterized by the ectopic presence of bone tissue in the soft tissue leading to severe loss of function in the extremities. Recent studies have indicated that immune cell infiltration and inflammation are involved in aberrant bone formation. In this study, we found increased monocyte/macrophage and mast cell accumulation during early HO progression. Macrophage depletion by clodronate liposomes and mast cell stabilization by cromolyn sodium significantly impeded HO formation. Therefore, we proposed that the dietary phytochemical quercetin could also suppress immune cell recruitment and related inflammatory responses to prevent HO. As expected, quercetin inhibited the monocyte-to-macrophage transition, macrophage polarization, and mast cell activation *in vitro* in a dose-dependent manner. Using a murine burn/tenotomy model, we also demonstrated that quercetin attenuated inflammatory responses and HO *in vivo*. Furthermore, elevated SIRT1 and decreased acetylated NFκB p65 expression were responsible for the mechanism of quercetin, and the beneficial effects of quercetin were reversed by the SIRT1 antagonist EX527 and mimicked by the SIRT agonist SRT1720. The findings in this study suggest that targeting monocyte/macrophage and mast cell activities may represent an attractive approach for therapeutic intervention of HO and that quercetin may serve as a promising therapeutic candidate for the treatment of trauma-induced HO by modulating SIRT1/NFκB signaling.

**Graphical Abstract:**
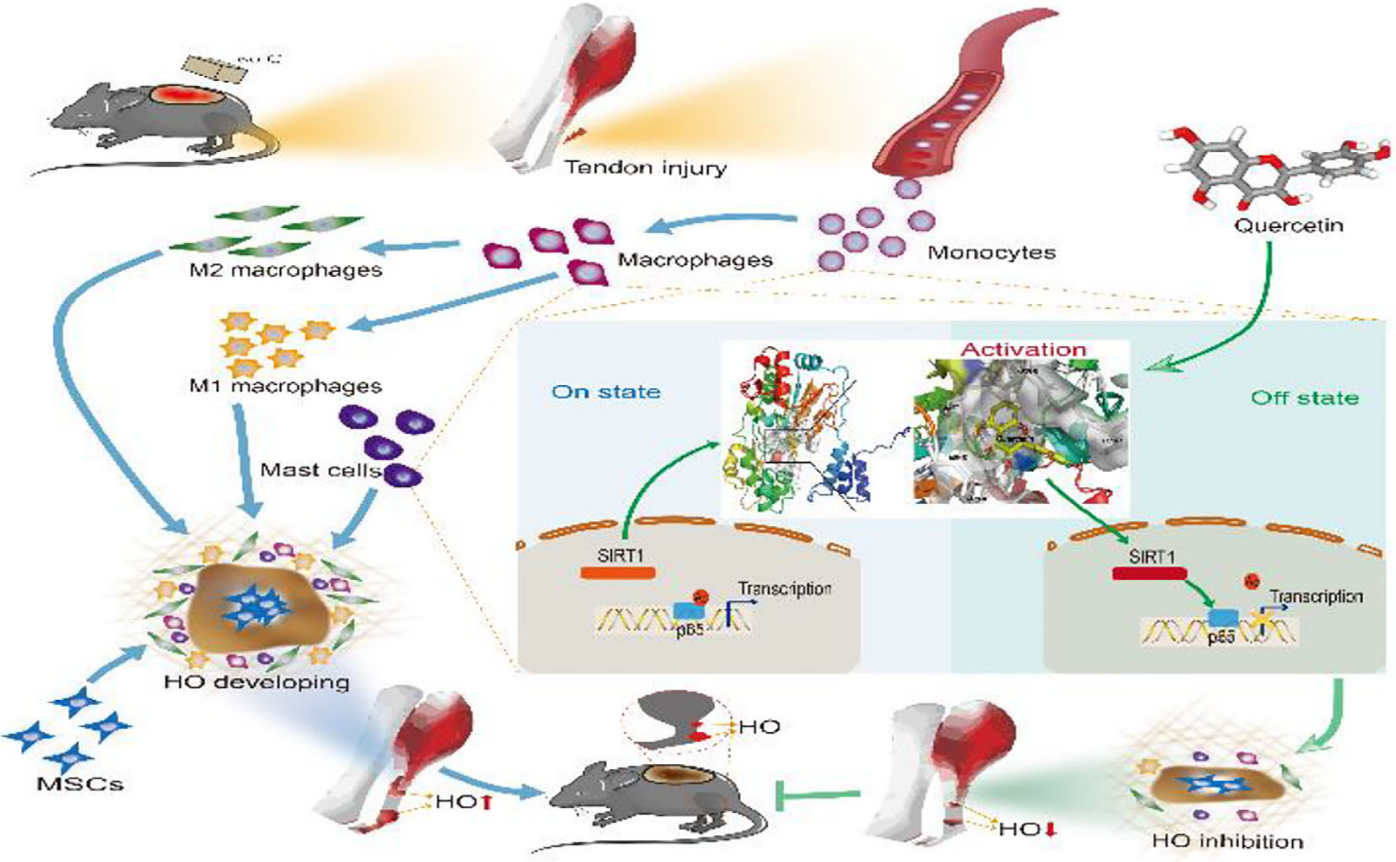


## Introduction

Heterotopic ossification (HO) is abnormal bone formation in soft tissue following trauma, including severe elbow fracture, total joint arthroplasty and even brain injury, which is an annoying and debilitating comorbidity affecting patients’ daily activity and quality of life (1, 2) HO is implicated in 65-91% of combat-related injuries (3, 4), 1.5-8% of burn injuries (5), and 13.6% of severe brain injuries (6) and is responsible for more than 30% of musculoskeletal consultations (7), imposing a heavy health burden on society and a cost burden on the economy. Surgical HO resection either by arthroscopy or open arthrolysis is currently the primary treatment for HO when mature bone is found, while the long-term clinical results remain to be evaluated (8, 9). Because there is still a relatively high recurrence rate after surgery (10, 11), full understanding of the complex mechanism underlying the occurrence of HO and the application of effective and reliable pharmacological prophylaxis is urgently needed.

Macrophages and mast cells are both important players in innate immunity and inevitably participate in the pathological progression of many diseases in immune-competent animals, which has been validated in musculoskeletal disorders (12, 13). In the general pathological process of HO, multipotent cells unexpectedly accumulate in the soft tissue lesion region and differentiate into mature bone cells through an endochondral ossification process under relevant induction signals. This aberrant permissive microenvironment formation, according to our current knowledge, is often initiated and aggravated by inflammatory cues (14). Upon injury, circulating monocytes from peripheral blood respond and infiltrate the injury site, where they undergo the monocyte-to-macrophage transition and polarization, secreting a plethora of proinflammatory mediators, such as tumor necrosis factor alpha (TNF-α), interleukin (IL)-1β, IL-6, monocyte chemoattractant protein-1 (MCP-1) and transforming growth factor β (TGF-β), creating an osteogenesis-promoting niche (15, 16). Mast cells also engender the inflammatory microenvironment by activating and inducing inflammatory cascades, thereby recruiting more monocytes and osteoprogenitors, leading to HO formation (12, 17). Targeting monocyte/macrophage and mast cell inflammatory responses thus seems to be a promising strategy for HO inhibition.

Sirtuin 1 (SIRT1) is a conserved nicotinamide adenosine dinucleotide (NAD)-dependent mammalian protein deacetylase with diverse biological functions. SIRT1 is generally delineated to perform functions in aging, metabolism and the cell cycle (18–20). In recent years, an increasing number of researchers have also reported that SIRT1 can coordinate inflammatory signaling and be viewed as a seminal target for immune microenvironment modulation. In a study with a murine hepatic ischemia/reperfusion injury model (21), SIRT1 activation alleviated leukocyte infiltration, and higher SIRT1 levels were associated with a lower proinflammatory cytokine profile. In a model of osteolysis (22), SIRT1 activation by hydrogen sulfide alleviated the particle-induced inflammatory response and prevented bone resorption. In addition, SIRT1 agonists were capable of blunting Th17 polarization and dampening IL17A production in metastatic colon cancer patients (23).

Despite numerous efforts devoted to the development of pharmacotherapy for HO, most existing therapeutic modalities have failed. Although several nonsteroidal anti-inflammatory drugs, such as indomethacin and celebrex, have shown some efficacy in clinical practice, the accompanying side effects cannot be ignored (24–26). In recent decades, the widespread discovery of the bioactivity of natural herbs has illuminated the potential use of natural compounds in HO therapy.

Quercetin is a natural flavonoid class of polyphenols commonly found in various fruits and vegetables, including blueberries, broccoli, onions, and green tea, and has recently been investigated by many researchers to alleviate diseases characterized by altered immune responses. Vanessa et al. (27) indicated that quercetin exposure reduced the activation and cytokine production of bone marrow-derived dendritic cells, while Tan et al. (28) demonstrated that quercetin strongly attenuated cisplatin-induced kidney inflammation and functional injury. By virtue of the natural compound properties of quercetin, it is also reasonable to predict that quercetin can exert its pharmacological functions with minimal side effects. This was corroborated by a study performed by Pereira et al. (29), who detected the genoprotective and cytoprotective effects of quercetin, making quercetin a promising drug for clinical application. However, whether quercetin can inhibit trauma-induced HO in an immune response-dependent manner remains to be determined.

In this study, we hypothesized that quercetin could attenuate trauma-induced HO through immune response regulation by modulating SIRT1 signaling. Our results revealed that SIRT1 might mediate the underlying mechanism of dysregulated immune responses during HO progression. We further demonstrated that quercetin with high SIRT1 binding affinity possessed an excellent anti-ossification effect and relieved macrophage and mast cell infiltration in a murine burn/tenotomy model. In vitro, quercetin successfully restrained monocyte-to-macrophage transition, ameliorated macrophage polarization and disabled mast cell degranulation.

## Materials and Methods

### Reagents and Materials

Quercetin, SRT1720 HCI (SIRT1 agonist) and EX527 (SIRT1 antagonist) were purchased from Selleck Chemicals (Houston, TX, USA). Phorbol myristate acetate (PMA), lipopolysaccharide (LPS) and cromolyn sodium were purchased from Sigma Aldrich (St. Louis, MO, USA). Clodronate liposomes were obtained from liposoma B. V (Netherlands). Fetal bovine serum (FBS) was purchased from Gibco (Carlsbad, CA, USA). RPMI 1640 basal culture medium and penicillin/streptomycin were provided by HyClone (Logan, UT, USA). Anti-SIRT1 (Cat# ab189494), anti-TGF-β1 (Cat# ab92486), and anti-tryptase (Cat# ab2378) antibodies were purchased from Abcam Biotechnology (Cambridge, MA, USA). Anti-PDGFRα (Cat# 3174) antibody was purchased from Cell Signaling Technology (CA, USA). Anti-NFkB p65 (acetyl Lys310) antibody (Cat# GTX86963) was purchased from Genetex (CA, USA). Anti-CD206 antibody (Cat# AF2535) was provided by R&D Systems (MN, USA). Anti-β-actin antibody (Cat# 66009-1-Ig) was purchased from Proteintech (Chicago, IL, USA). Anti-IL-1β (Cat# GB11113), anti-TNF-α (Cat# GB13188-2), anti-IL-6 (Cat# GB11117), anti-MCP-1 (Cat# GB11199), anti-F4/80 (Cat# GB11027), anti-F4/80 (GB11119) and anti-IL-10 (Cat# GB11108) antibodies were obtained from Servicebio (Wuhan, China).

### Cell Culture

Bone marrow-derived macrophages (BMDMs) were isolated from the bone marrow of 8- to 10-week-old C57BL/6 mice as previously described with minor modifications (30). Briefly, both the femur and tibia were excised with soft tissue completely removed, and then the bone marrow cells were collected by flushing the marrow cavity using a 26G needle. The harvested cells were incubated in RPMI 1640 complete culture medium in the presence of 40 ng/ml of macrophage colony-stimulating factor (M-CSF), and the medium was refreshed after 3 days and incubated for another 3 days until macrophage maturation. For experiments, BMDMs were collected by scraping and cultured in RPMI 1640 complete culture medium supplemented with 20 ng/ml of M-CSF.

THP-1 and P815 cell lines were purchased from the Cell Bank of the Chinese Academy of Sciences. THP-1 and P815 cells were maintained in RPMI 1640 complete culture medium supplemented with 10% fetal bovine serum and 1% penicillin/streptomycin. All cells were cultured at 37°C in a 5% CO_2_ humidified atmosphere.

### Cell Viability Assay

The effect of quercetin on the viability of the three cell lines was tested by CCK-8 (Dojindo Molecular Technologies, Tokyo, Japan) as previously described. Briefly, THP-1 cells at 3×10^5^/well, BMDMs at 10^5^/well, or P815 cells at 2×10^5^/well were seeded in a 96-well plate and cultured overnight. The next day, quercetin solution at a gradient concentration (μM) was added, and the cells were further incubated for 24 h or 48 h. Ten microliters of CCK8 solution was added to each well at the endpoint of each experiment. After another 2 h incubation, the absorbance of each well was examined at 450 nm by a SpectraMax M3 microplate reader (Molecular Devices, Sunnyvale, USA).

### Cell Migration Assay

The cell migration assay was performed in Transwell chambers with a membrane pore size of 8 μm as previously described (31). Briefly, BMDMs were inoculated into the upper chamber of a Transwell apparatus (Corning, USA) and treated with EX527 or SRT1720 HCI for 6 h and quercetin for 1 h. Then, the BMDMs were incubated in serum-free medium and allowed to migrate toward the serum-supplemented medium supplemented with MCP-1 (2 ng/ml) through the chamber membrane for 24 h. Then, the cells were fixed with 4% paraformaldehyde and stained with 0.5% crystal violet for 20 min. The cells in the upper surface of the chamber were removed, and the cells that migrated to the bottom surface were photographed by a Nikon ECLIPSE *Ts2R*-FL fluorescence microscope (Tokyo, Japan). Six random fields from each well were selected to determine the average number of migrated cells.

### Monocyte-to-Macrophage Transition Observation

THP-1 cell morphological changes and adhesion rates after PMA stimulation were assessed by crystal violet staining. THP-1 cells were inoculated at a density of 4×10^5^/well in a 6-well plate and pretreated with EX527 or SRT1720 HCI for 6 h and quercetin for 1 h. After incubation with PMA in the presence or absence of quercetin, EX527 or SRT1720 HCI for 48 h, attached cells were fixed with 4% paraformaldehyde before staining with 0.5% crystal violet staining solution for 20 min. Stained cells were washed three times in phosphate buffer solution (PBS), and microscopy images were taken using a Nikon ECLIPSE *Ts2R*-FL fluorescence microscope (Tokyo, Japan). Ten percent acetic acid was used to elute the stained cells, which were then transferred into a 96-well plate. Absorbance was recorded at 595 nm with a spectrophotometer.

### Cell Immunofluorescence Staining

After treatment, cells were fixed in 4% paraformaldehyde followed by permeabilization using 0.5% Triton X-100. BSA (1%) was used to block the cells, and the cells were incubated with anti-CD206 or anti-INOS antibodies at 4°C. Conjugated secondary antibodies were then employed to stain the cells in the dark. The cell nuclei were counterstained with 4,6-diamidino-2-phenylindole (DAPI), and cells were photographed using a digital slide scanner (Pannoramic MIDI; 3DHISTECH Ltd).

### Flow Cytometry Analysis

Flow cytometry analysis was performed to examine the surface marker changes in THP-1 cells or BMDMs. THP-1 cells or BMDMs were seeded in a 6-well plate and treated with the respective compounds. Then, the cells were harvested with a cell scraper and washed with PBS three times. The acquired cells were blocked with CD16/32 antibodies (Clone 93, Biolegend) for 15 min on ice, and then PE-CD11b (Clone ICRF44, Biolegend), FITC-CD14 (Clone 63D3, Biolegend), BV421-F4/80 (Clone BM8, Biolegend), PE-Dazzle594-CCR7 (Clone 4B12, Biolegend), and PE-MMR (Clone C068C2, Biolegend) antibodies were applied to stain the cells for 30 min in the dark. Following washing in PBS, cell marker expression was assayed using FACS AriaIII (BD, New Jersey, USA), and the data were analyzed by FlowJo software (Tree Star Inc., San Carlos, USA).

### Real-Time Quantitative Polymerase Chain Reaction

Total RNA was isolated using TRIzol reagent (Invitrogen) following the manufacturer’s instructions. Reverse transcription was performed using M-MLV reverse transcriptase (Takara) to synthesize complementary DNA. SYBR Green Premix Ex Taq (Takara) was applied to quantify the target gene mRNAs. The gene primers used are listed in **Supplementary Table 1** with GAPDH as a housekeeping gene.

### Western Blot Analysis

Western blot analysis was performed as previously described (28). Specifically, whole cells or tissues were lysed in RIPA lysis buffer (Epizyme, Shanghai) supplemented with a proteinase inhibitor cocktail (Epizyme, Shanghai) on ice. Protein in the supernatant was collected by centrifugation, and the protein concentration was determined by BCA assay. Equal amounts of protein (30 µg) were loaded onto sodium dodecyl sulfate-polyacrylamide electrophoresis gels. Separated proteins in gels were transferred to polyvinylidene fluoride membranes. Subsequently, the membranes were blocked in 5% nonfat milk or bovine serum albumin (BSA) for 1 h and probed with primary antibodies at 4°C overnight. Following incubation with HRP-conjugated secondary antibodies for 1 h at room temperature, enhanced chemiluminescence reagent (Epizyme, Shanghai) was applied to develop the signal, which was detected by a ChemiDoc CRS imaging system (Bio-Rad, USA).

### Enzyme-Linked Immunosorbent Assay (ELISA)

The secretion levels of the cytokines TNF-α, IL-1β, MCP-1, IL-6, TGF-β and IL-10 were examined using ELISA kits (Anogen) in accordance with the manufacturer’s instructions. The concentrations of histamine and tryptase released by the P815 cells were also assessed using ELISA kits provided by Fitzgerald (Birmingham, United Kingdom) according to the manufacturer’s instructions.

### Animals and Burn/Tenotomy Murine Model Establishment

Eight- to ten-week-old male C57BL/6 mice raised under specific pathogen-free conditions were housed in the animal experimental center of Shanghai Sixth People’s Hospital. The mice were given access to food and water ad libitum. Before modeling, mice were acclimated to the environment for at least 1 week. Trauma-induced HO was created using a murine burn/tenotomy model previously reported by Peterson et al. (32). After anesthesia with 1% pentobarbital sodium, a 0.5 cm longitudinal incision was made along the medial aspect of the distal hindlimb. The Achilles tendon was found and transected at the midpoint without stitching, and then the incision was closed with a 5-0 Vicryl stitch. After Achilles tenotomy, a concomitant approximately 30% body surface burn injury was made with a 2 cm x 2 cm x 3 cm aluminum block weighing 35 g. The block was heated to 60°C in a water bath before being placed on the shaved dorsal skin and maintained for 17 sec. Sham surgery was performed by simply exposing the Achilles tendon and then closing the incision without burn injury. For macrophage depletion, clodronate liposomes (1.33 ml/100 g) were intraperitoneally injected 2 days prior to surgery. At the time of surgery, 100 µl of clodronate liposomes was delivered directly into the tenotomy site, and then intraperitoneal injection (1.33 ml/100 g) was maintained thereafter weekly for 2 weeks. For mast cell stabilization, cromolyn sodium (10 mg/kg/day) was intraperitoneally injected every 2 days for 2 weeks, beginning from 2 days before surgery. Mice in the Sham and Positive groups received PBS that was administered using the same strategy.

To assess the effect of quercetin on HO, animals were randomly divided into 4 groups: Sham group (sham surgery with vehicle), Positive group (burn/tenotomy with vehicle), Low group (burn/tenotomy with a low dose of quercetin), High group (burn/tenotomy with a high dose of quercetin). Low and high doses of quercetin were intraperitoneally injected at doses of 20 mg/kg/d and 50 mg/kg/d daily for 2 weeks from the day of surgery. Mice in the Sham and Positive groups received 4% dimethyl sulfoxide (DMSO) solution that was administered using the same strategy.

To evaluate the role of SIRT1 in the effectiveness of quercetin, animals were randomly divided into 5 groups: sham group (sham surgery with vehicle), positive group (burn/tenotomy with vehicle), Q group (burn/tenotomy with quercetin), Q+E group (burn/tenotomy with quercetin and EX527), and SRT group (burn/tenotomy with SRT1720 HCI). Mice in the Q and Q+E groups received quercetin (50 mg/kg/d, i.p.) in the presence or absence of EX527 (5 mg/kg/d, i.p.) for 2 weeks from the day of surgery. Mice in the SRT group received SRT1720 HCI (25 mg/kg, i.p.) every other day for 2 weeks from the day of surgery. Mice in the Sham and Positive groups received 4% DMSO solution that was administered using the same strategy.

All animal experimental protocols described above were approved by the Institutional Animal Care and Use Committee (IACUC) of the Shanghai Sixth People’s Hospital, and experimental procedures were conducted in strict accordance with the guidelines of the National Institutes of Health Guide for the Care and Use of Laboratory Animals. All reports on animal studies complied with the ARRIVE guidelines.

### Histological Observations

Animals were euthanized at 3 days, 7 days, 3 weeks or 10 weeks for histological staining and observation. Skin was carefully removed from the distal hindlimb, soft tissues from the convergence site of the calf muscle and Achilles tendon to the tendon calcaneus insertion site were excised and slightly manicured. After fixation in 10% (v/v) formalin, tissues were decalcificated with 19% ethylenediaminetetracetic acid (EDTA) solution when the sampling time point after surgery was larger than 6 weeks. Tissues were then subjected to standardized dehydration procedures through graded ethanol and embedded in paraffin. Longitudinal sections were made from the harvested tissue, and an approximate thickness of 5 microns was selected. Sections were mounted onto Thermo Superfrost^®^ Plus slides. Routine histological, immunohistochemistry or immunofluorescence staining was carried out to analyze the changes in tissue morphology and composition as previously described (33).

### Micro-CT Scanning

Mice at week 10 after burn/tenotomy were sacrificed, and hindlimbs from each group were collected in 10% (v/v) formalin, fixed for 48 h and then analyzed by the high-resolution Micro-CT scanner Skyscan 1176 (software=Version 1.1 (build 6), Bruker, Kontich, Belgium). The parameter was set at 18 mm for isometric resolution and 70 kV for voltage. Three-dimensional images were reconstructed and obtained from CTvox software (Version). Bone volume calculation was finished using CTan software (Version 1.15.4.0+, Bruker) as previously described (34), and the high-density mass in the soft tissue with a Hounsfield unit above 272 was considered to be heterotopic bone.

### Homology Modeling and Molecular Docking

To predict a possible direct interaction between the small molecule quercetin and the active site of SIRT1 and find their preferred binding site, computer molecular docking was applied using SRT1720 as a reference drug. Since the RCSB Protein Data Bank (PDB) did not provide the structure of mouse SIRT1, we downloaded the amino acid sequence of SIRT1 from the UniProtKB database (http://www.UniProt.org/) and performed homology modeling with SWISS-MODEL (https://www.swissmodel.expasy.org/) to obtain the structure of SIRT1 based on the structure of NAD-dependent protein deacetylase sirtuin-1 (PDB: 4KXQ). PROCHECK was used to examine the stereochemical quality of the structure obtained from SWISS-MODEL to draw the Ramachandran plot. The interactions between SIRT1 activated pocket (ARG266, SER267, GLN337, SER433, SER434) and quercetin or SRT1720 simulated by AutoDocking vina, default AutoDocking vina parameters were used. Multiple poses and binding interaction geometries of quercetin or SRT1720 to SIRT1 were estimated, and the respective conformation energies were scored. Finally, a molecular operating environment (PyMOL) was used to visualize the protein-ligand complexes.

### Statistical Analysis

Data analysis was performed in GraphPad Prism 8. Data are expressed as the mean ± standard deviation (SD). Data were checked for normality by Shapiro-Wilk test, and homogeneity of variance was determined by one-way ANOVA. Comparisons among groups were accomplished using one-way or two-way ANOVA followed by Tukey’s test for *post hoc* comparisons when data were normally distributed, wherein two-way ANOVA was performed when two independent variables were required to be analyzed. Kruskal-Wallis tests were performed when data were not normally distributed. Comparisons of categorial data were accomplished using the chi-square test. Statistical significance was set at P<0.05, and two-tailed tests were conducted. Prior sample sizes were determined using PASS software version 15 (NCSS statistical software, UT) according to the results obtained from preliminary studies and set α=0.05 and β=0.1. All experiments were independently performed for at least three biological and technical replicates.

## Results

### Macrophages and Mast Cells Are Key Players in Trauma-Induced Heterotopic Ossification

**Figure 1:**
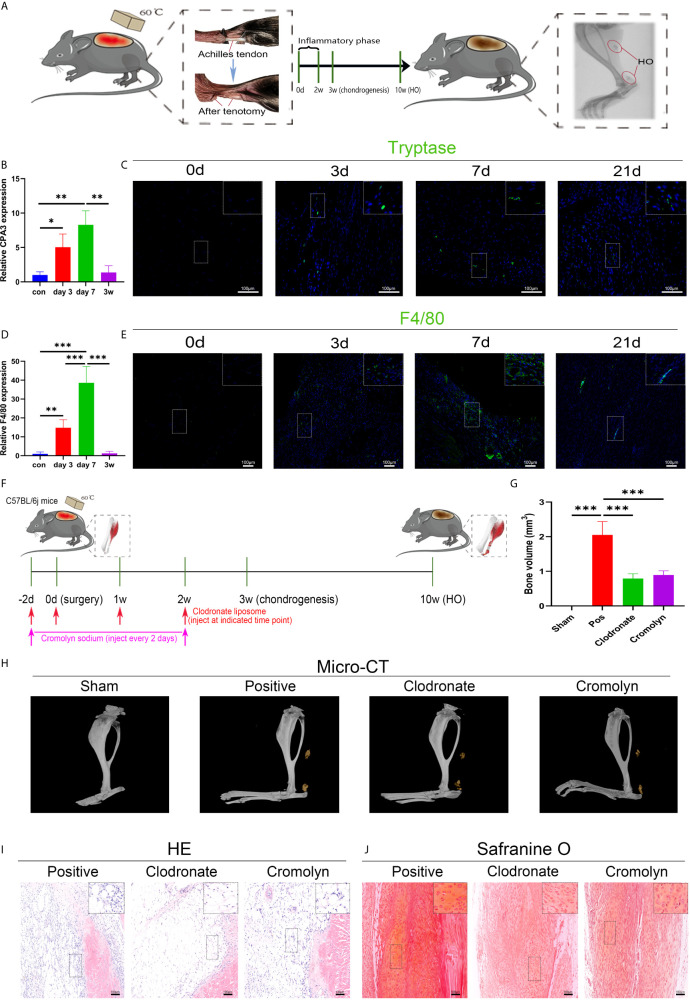
Both macrophages and mast cells are critical participants in trauma-induced heterotopic ossification (HO). **(A)** Schematic depiction of the establishment of a murine burn/tenotomy model and different stages of HO progression. **(B, D)** Relative gene expression of the mast cell marker CPA3 and macrophage marker F4/80 as evaluated by qRT-PCR. **(C, E)** Immunofluorescence staining of the mast cell marker tryptase and macrophage marker F4/80 at the indicated times. **(F)** Schematic depiction of macrophage depletion and mast cell stabilization protocols for animal experiments. **(G, H)** Micro-CT scanning for observation and quantification of HO formation. **(I)** HE staining of the tissue section 3 days after burn/tenotomy. **(J)** Safranine O staining of the tissue section 3 weeks after burn/tenotomy. Original magnification is 40x for tryptase fluorescence staining and 20x for other staining. Inserts are approximately 3.5x magnified images of the boxed area. N=4/group, *P < 0.05, **P < 0.01, ***P < 0.001.

A murine burn/tenotomy model was established according to the literature (32). The role of macrophages and mast cells in trauma-induced heterotopic ossification was confirmed as previously reported (35–38) (**Figure 1A**). F4/80 was used as a marker of macrophages, and CPA3 or tryptase was used as a marker of mast cells. Both qRT-PCR and immunofluorescence staining showed that macrophages and mast cells appeared early at 3 days, peaked at 7 days, and persisted for up to 3 weeks (**Figures 1B–E**). To investigate the contributions of macrophages and mast cells at early stages to trauma-induced HO, macrophage depletion and mast cell stabilization were performed for 2 weeks using clodronate liposomes and cromolyn sodium, respectively (**Figure 1F**). As revealed by micro-CT, either clodronate liposomes or cromolyn sodium successfully diminished HO formation (**Figures 1G, H**). Concordantly, when examined at early stages of HO progression, we found that both inflammatory cell infiltration and chondrogenesis were prominently blocked by clodronate liposomes or cromolyn sodium (**Figures 1I, J**), indicating that the two treatments reshape early events during HO progression.

### The SIRT1/NFκB Pathway Was Involved in Altered Immune Responses to Trauma-Induced Heterotopic Ossification

**Figure 2:**
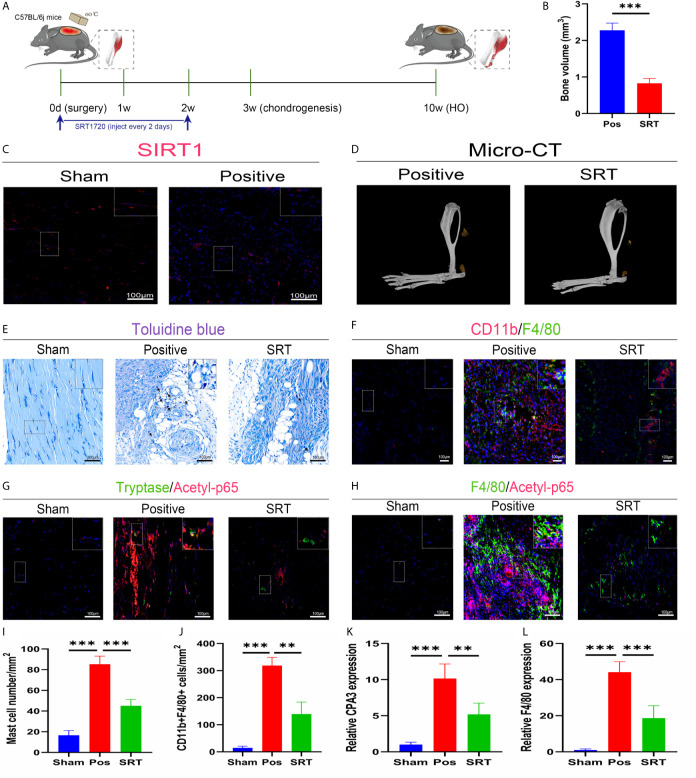
SIRT1/NFκB signaling was responsible for the dysregulated immune responses in trauma-induced HO. **(A)** Schematic depiction of the agonist treatment protocols for animal experiments. **(B, D)** Observation and quantification of HO formation using micro-CT. **(C)** Fluorescence staining of SIRT1 7 days after burn/tenotomy. **(E)** Toluidine blue staining for observation of mast cells 7 days after burn/tenotomy. **(F)** Double fluorescence staining of CD11b and F4/80 7 days after burn/tenotomy. **(G)** Double fluorescence staining of tryptase and acetyl-p65 7 days after burn/tenotomy. **(H)** Double fluorescence staining of F4/80 and acetyl-p65 7 days after burn/tenotomy. **(I)** Quantification of the mast cells in toluidine blue staining images. **(J)** Quantification of CD11b+F4/80+ cells in double fluorescence staining images. **(K)** Relative gene expression of the mast cell marker CPA3 as determined by qRT-PCR. **(L)** Relative gene expression of macrophage marker F4/80 as determined by qRT-PCR. Original magnification is 40x. Inserts are approximately 3.5x magnified images of the boxed area. N=4/group. ***P < 0.001.

To explore the underlying molecular mechanisms involved in these aberrant immune responses, we checked SIRT1 expression in trauma-induced HO according to a previous report on the immunomodulatory role of SIRT1. As expected, SIRT1 expression was suppressed 7 days after burn/tenotomy (**Figures 2A, C**). Based on this finding, we then examined whether SIRT1 activation benefits HO blockade. The results showed that direct activation of SIRT1 by SRT1720 HCI significantly hindered HO formation (**Figures 2B, D**). Moreover, decreased infiltration of monocyte-derived macrophages and mast cell infiltration was observed at early stages of HO by direct activation of SIRT1 by SRT1720 HCI (**Figures 2E, F, I, J**), confirming the involvement of SIRT1 in the molecular events underlying the immune response after injury. Downstream of SIRT1, we next evaluated the status of NFκB signaling, which is a master regulator of immune responses. As displayed by immunofluorescence staining, acetylated p65 expression was dramatically increased after trauma but inhibited during SIRT1 activation (**Figures 2G, H**). Correspondingly, reduced expression of CPA3 and F4/80 was also observed after SRT1720 HCI application (**Figures 2K, L**). These results indicate that upregulated acetylated p65 expression is involved in dysregulated macrophage and mast cell responses in HO, which are SIRT1 dependent.

### Quercetin Modulated SIRT1/NFκB Signaling During Immune Responses

**Figure 3:**
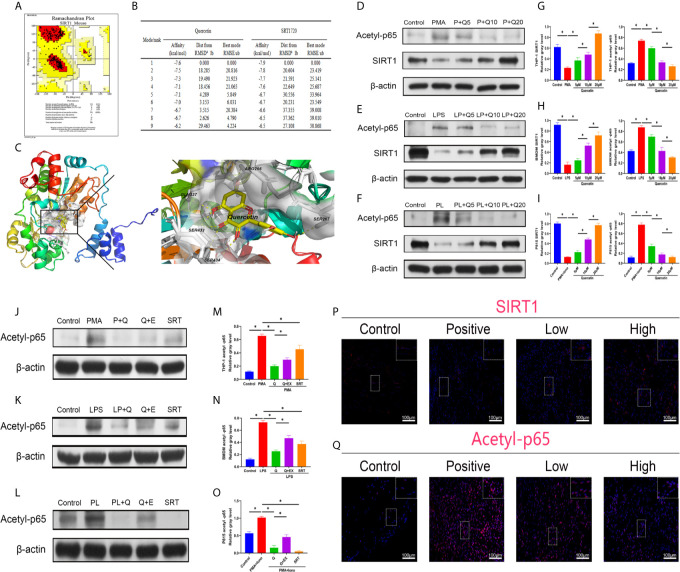
Quercetin targets and modulates SIRT1 to reduce NFκB acetylation during immune response modulation. **(A)** Ramachandran plot for homology-modeled mouse SIRT1. The core area was colored red and involved 94.2% of the amino acid residues, while the allowed area was colored yellow and involved 5.8% of the amino acid residues. None of the amino acid residues were located in the generous (light yellow) or disallowed (white) areas. **(B)** Tables listing the nine top binding affinities of quercetin or SRT1720 to SIRT1. Affinity denotes binding energy, RMSD represents root-mean-square deviation and RMSE represents root-mean-square error. **(C)** Binding and interaction sites of quercetin with SIRT1 are shown. Cells were pretreated with 5 μM, 10 μM, or 20 μM quercetin for 1 h, and then incubated with respective stimulus for 1 h or 24 h. The effects of quercetin on SIRT1 and acetylated p65 expression in **(D)** THP-1, **(E)** BMDMs and **(F)** P815 cells during their activation were assessed by western blot. The relative level of SIRT1 and acetyl-p65 in **(G)** THP-1, **(H)** BMDMs and **(I)** P815 cells quantified using ImageJ and normalized to β-actin are shown. Cells were pretreated with 20 μM quercetin for 1 h, 10 μM EX527 and 1 μM SRT1720 HCI for 6 h and then incubated with respective stimulus for 1 h or 24 h. The effects of SIRT1 activation and inhibition on acetylated p65 expression in **(J)** THP-1, **(K)** BMDMs and **(L)** P815 cells during their activation were assessed by western blot. The relative level of acetylated p65 in **(M)** THP-1, **(N)** BMDMs and **(O)** P815 cells quantified using ImageJ and normalized to β-actin is shown. **(P, Q)** Immunofluorescence staining showing the expression of SIRT1 and acetylated p65 in the tissues 7 days after tenotomy. Original magnification is 40x. Inserts are approximately 3.5x magnified images of the boxed area. N=4/group. *P < 0.05.

To better apply the SIRT1-targeting benefits to trauma-induced HO, we tested the treatment efficacy of the natural chemical quercetin. First, we confirmed the direct SIRT1 activation capacity of quercetin. Through a computer molecular docking analysis, we showed that quercetin has high binding affinity to mouse SIRT1. The homology-modeled structure of mouse SIRT1 met the criteria for the probability density function of energy, as revealed by the Ramachandran plot test (**Figure 3A**). Docking analysis showed high binding affinities of quercetin to SIRT1, ranging from -7.6 to -6.2, were comparable to those of SRT1720, ranging from -7.9 to -6.5 (**Figure 3B**). Using PyMOL, quercetin was observed to interact with the SIRT1-activated pocket (ARG266, SER267, GLN337, SER433, SER434) in hydrogen bonds (**Figure 3C**). These results indicate that quercetin may be a perfect ligand for directly interacting with and activating SIRT1. In addition, western blot analysis indicated that although attenuated expression of SIRT1 was found during the classical immune response, including the monocyte-to-macrophage transition, M1 polarization, and mast cell activation, quercetin largely restored SIRT1 expression, suggesting that SIRT1 protein expression can also be modulated by quercetin (**Figures 3D–I**).

Along with the reduction in SIRT1, the expression of acetylated NFκB p65 was increased during the processes of monocyte-to-macrophage transition, M1 polarization, and mast cell activation, whereas quercetin addition was significantly inhibitory (**Figures 3D–I**). Furthermore, cotreatment with SIRT1 antagonist EX527 significantly restored the impaired acetylated p65 expression by quercetin, while SIRT1 agonist SRT1720 HCI abrogated the acetylated p65 expression raised by the abovementioned stimulants, verifying the causal relationship between SIRT1 activation and acetylation of NFκB p65 (**Figures 3J–O**). In vivo, the decreased immunofluorescence staining of SIRT1 and enhanced staining of acetyl-p65 were reversed by quercetin (**Figures 3P–Q**).

### Quercetin Reduced Monocyte-to-Macrophage Transition and Macrophage Polarization *In Vitro*

**Figure 4:**
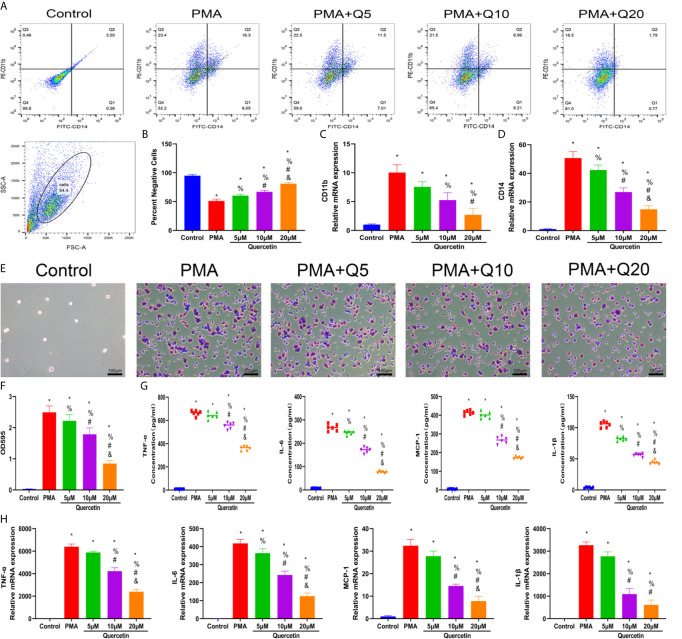
Quercetin inhibited the monocyte-to-macrophage transition *in vitro*. THP-1 cells were pretreated with quercetin for 1 h and then incubated with PMA for 48 h. **(A)** Flow cytometry analysis for CD14 and CD11b. **(B)** Bar graphs illustrating the percentage of CD14 and CD11b double-negative cells. **(C, D)** The relative gene expression of CD11b and CD14 was determined using qRT-PCR. **(E)** Crystal violet staining of transitioned macrophages from THP-1 cells. **(F)** OD595 value of eluted crystal violet staining. **(G)** Inflammatory cytokine secretion during the monocyte-to-macrophage transition was examined by ELISA. THP-1 cells were pretreated with quercetin for 1 h and then incubated with PMA for 24 h. **(H)** The relative gene expression of inflammatory cytokines was tested by qRT-PCR. *P < 0.05 compared with the control group, ^%^P < 0.05 compared with the PMA group, ^#^P < 0.05 compared with the 5 μM group, ^&^P < 0.05 compared with the 10 μM group.

The effect of quercetin on immune responses was first evaluated *in vitro*. Monocyte-to-macrophage transition represents an important etiology of local macrophage infiltration after musculoskeletal injury; thus, the effect of quercetin on monocyte-to-macrophage transition was assessed. A CCK-8 assay was first conducted to determine and exclude cytotoxic quercetin concentrations for fear that toxicological effects of quercetin could be mistaken as pharmacological effects. Noncytotoxic concentrations of quercetin on THP-1 cells were below 20 μM within 48 h (**Supplementary Figure 1A**). Flow cytometry analysis and qRT-PCR revealed that quercetin decreased the expression of the human macrophage surface markers CD11b and CD14 in THP-1 cells in a dose-dependent manner after quercetin supplementation compared with that stimulated with PMA (**Figures 4A–D**). Consistent with this, crystal violet staining verified the reduced cell adhesion and pseudopod formation, indicating decreased monocyte-to-macrophage transition (**Figures 4E, F**). In addition, the accompanying production of inflammatory cytokines, including TNF-α, IL-1β, IL-6 and MCP–1, during monocyte-to-macrophage transition was significantly downregulated by quercetin (**Figures 4G, H**).

**Figure 5:**
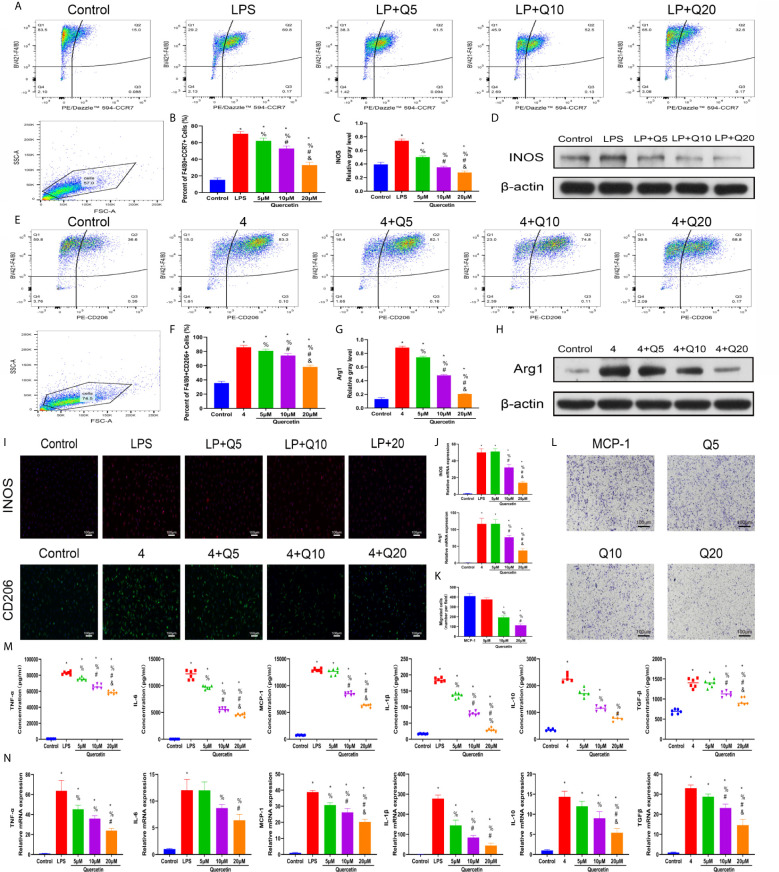
Quercetin impaired macrophage polarization and migration *in vitro*. BMDMs were pretreated with quercetin for 1 h and then incubated with LPS or IL-4 for 24 h. **(A)** Flow cytometry analysis for F4/80 and CCR7. **(B)** Bar graphs illustrating the percentage of F4/80 and CCR7 double-positive cells. **(C, D)** The protein expression of INOS was detected by western blot. **(E)** Flow cytometry analysis for F4/80 and CD206. **(F)** Bar graphs illustrating the percentage of F4/80 and CD206 double-positive cells. **(G, H)** The protein expression of Arg1 was detected by western blot. **(I)** Immunofluorescence staining of INOS or Arg1. **(J)** The relative gene expression of INOS or Arg1 was analyzed using qRT-PCR. **(K, L)** Crystal violet staining of migrated BMDMs after the Transwell assay. **(M)** Inflammatory cytokines in the supernatants of BMDMs upon different treatments. BMDMs were pretreated with quercetin for 1 h and then incubated with LPS or IL-4 for 8 h. **(N)** The relative gene expression of inflammatory cytokines was detected by qRT-PCR. *P < 0.05 compared with the control group, ^%^P < 0.05 compared with the LPS or 4 group, ^#^P < 0.05 compared with the 5 μM group, ^&^P < 0.05 compared with the 10 μM group.

As macrophage polarization has been validated to profoundly affect wound healing, we investigated the effects of quercetin on macrophage polarization. We chose drug concentrations below 40 μM because no significant toxicity was found for quercetin at these concentrations on BMDMs (**Supplementary Figure 1B**). A classical *in vitro* macrophage polarization cell model was created using LPS stimulation for M1 and IL-4 stimulation for M2. Flow cytometry analysis suggested that quercetin reversed BMDM polarization to either the M1 or M2 subtype in a dose-dependent manner (**Figures 5A, E**), which was corroborated by the qRT-PCR, western blot and immunofluorescence staining results, wherein the expression of the M1 marker INOS or the M2 marker CD206 was evidently decreased (**Figures 5B–D, F–J**). Next, the Transwell assay was performed using MCP-1 as a chemoattractant. Crystal violet staining revealed that quercetin significantly reduced the number of cells on the bottom surface of the membrane, indicating impaired migration (**Figures 5K, L**). To further determine the influence of quercetin on inflammatory cytokine production accompanying macrophage polarization, ELISA and qRT-PCR were performed. The gene expression and secretion of cytokines, including TNF-α, IL-1β, IL-6, MCP–1, IL-10 and TGF-β, were significantly upregulated upon LPS or IL-4 stimulation. However, the addition of quercetin restrained cytokine production at both the transcriptional and protein levels in a dose-dependent manner. (**Figures 5M, N**).

### Quercetin Ameliorated Mast Cell Activation *In Vitro*

**Figure 6:**
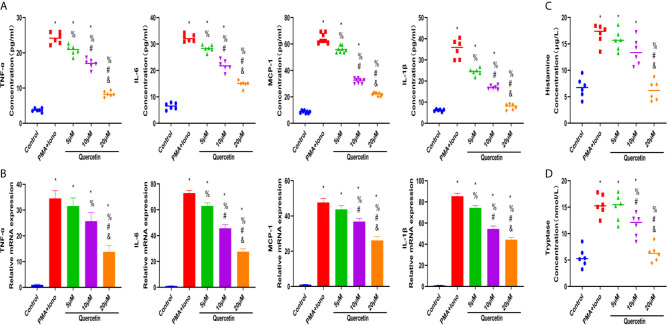
Quercetin disabled mast cell secretion of proinflammatory cytokines *in vitro*. P815 cells were pretreated with quercetin for 1 h and then incubated with PMA and ionomycin for 24 h. **(A)** Inflammatory cytokine secretion of P815 cells was examined by ELISA. P815 cells were pretreated with quercetin for 1 h and then incubated with PMA and ionomycin for 8 h. **(B)** Relative gene expression of inflammatory cytokines in P815 cells as evaluated by qRT-PCR. **(C)** Histamine release from P815 cells was evaluated by ELISA. **(D)** Tryptase release from P815 cells as evaluated by ELISA. *P < 0.05 compared with the control group, ^%^P < 0.05 compared with the PMA+Iono group, ^#^P < 0.05 compared with the 5 μM group, ^&^P < 0.05 compared with the 10 μM group.

To test the therapeutic effectiveness of quercetin on mast cell activation, we used the classical mast cell stimulators A23187 and PMA to synergistically activate P815 cells *in vitro*. Cytotoxicity assays suggested that P815 cells maintained normal growth for 24 h at quercetin doses up to 20 μM (**Supplementary Figure 1C**). ELISA showed that A23187 and PMA significantly, albeit not dramatically, induced the secretion of IL-1β, TNF-α, IL-6 and MCP-1 in mast cells, whereas quercetin limited secretion in a dose-dependent manner, with 20 μM quercetin reaching the maximum inhibitory effect (**Figure 6A**). Similar results were obtained with qRT-PCR, showing the inhibitory effect of quercetin on mast cell activation (**Figure 6B**). Regarding histamine and tryptase release from granules of P815 cells, quercetin at a dose of 20 μM also showed a significant inhibitory effect, although the dose-dependent effect was not prominent (**Figures 6C, D**).

### Quercetin Attenuated Trauma-Induced Heterotopic Ossification by Abolishing Inflammatory Responses

**Figure 7:**
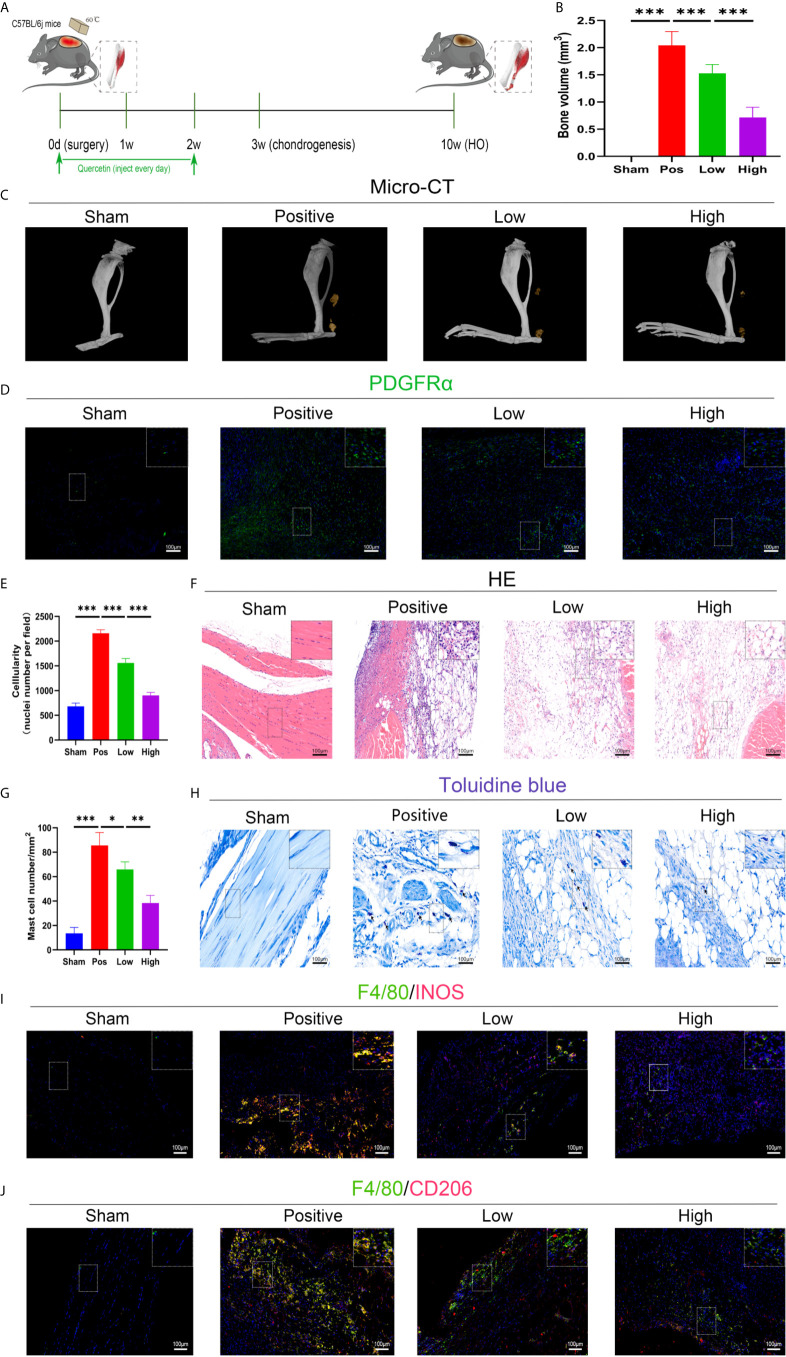
Quercetin effectively attenuated HO formation by diminishing macrophage and mast cell infiltration after tendon injury. **(A)** Schematic depiction of the quercetin treatment protocols for animal experiments. **(B, C)** Micro-CT quantification and observation of HO formation. **(D)** Immunofluorescence staining of the stem cell marker PDGFRα 7 days after burn/tenotomy. **(E, F)** HE staining for observation of tissue cellularity 3 days after burn/tenotomy. **(G, H)** Toluidine blue staining for observation of mast cell accumulation 7 days after burn/tenotomy. **(I)** Double fluorescence staining of F4/80 and INOS for observation of the M1 subtype of macrophages 7 days after burn/tenotomy. **(J)** Double fluorescence staining of F4/80 and CD206 for observation of the M2 subtype of macrophages 7 days after burn/tenotomy. Original magnification is 40x for toluidine blue staining and 20x for other staining. Inserts are approximately 3.5x magnified images of the boxed area. N=4/group, *P < 0.05, **P < 0.01, ***P < 0.001.

**Figure 8:**
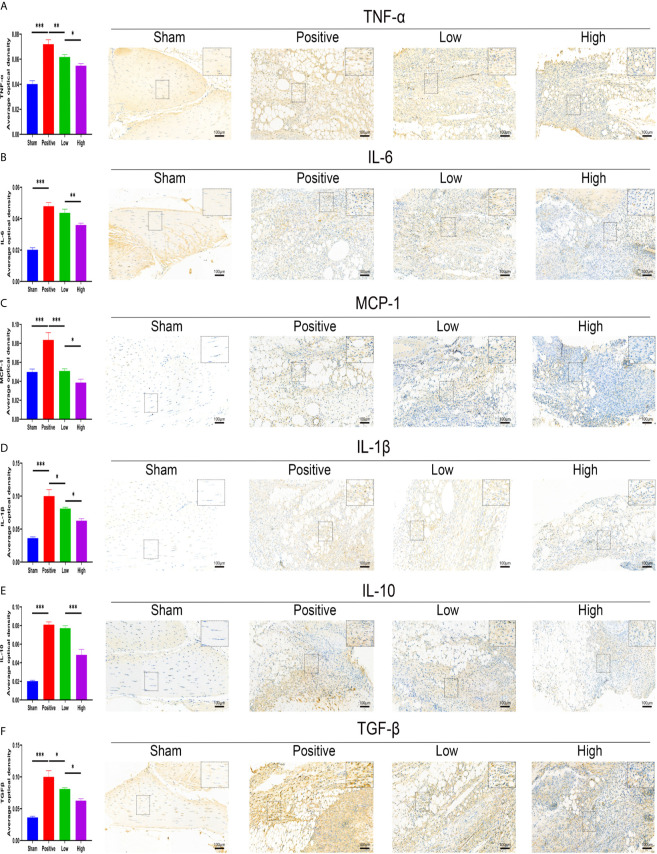
Quercetin relieved inflammatory mediator accumulation after tendon injury in a dose-dependent manner. Immunohistochemical staining and quantification of **(A)** TNF-α, **(B)** IL-6, **(C)** MCP-1, **(D)** IL-1β, **(E)** IL-10, and **(F)** TGF-β in tissue sections 7 days after burn/tenotomy. The average optical density was calculated using Image-Pro Plus software. Original magnification is 20x. Inserts are approximately 3.5x magnified images of the boxed area. N=4/group. *P < 0.05, **P < 0.01, ***P < 0.001.

The effect of quercetin on trauma-induced inflammatory responses and HO was then assessed in the murine burn/tenotomy model (**Figure 7A**). Micro-CT results confirmed that quercetin significantly inhibited trauma-induced HO, and this effect was dose-dependent (**Figures 7B, C**). Next, to assess whether there were changes in stem cell recruitment at earlier times, we determined whether PDGFRα+ cells were present, as previous studies identified PDGFRα as a common marker of osteoprogenitors in HO (39). The results of immunofluorescence staining revealed that PDGFRα+ osteoprogenitor accumulation was dramatically decreased after quercetin, with higher doses exerting a better effect (**Figure 7D**). To elucidate the above phenomenon, we examined the immune response at the early stage of HO progression. HE staining at 3 days post burn/tenotomy revealed that quercetin diminished tissue cellularity in a dose-dependent manner (**Figures 7E, F**). In particular, toluidine blue staining suggested that mast cell infiltration was significantly suppressed by quercetin (**Figures 7G, H**). Double fluorescence staining for F4/80 and INOS or F4/80 and CD206 was performed to identify M1 subtype and M2 subtype macrophages, respectively. The results showed that burn/tenotomy led to a dramatically increased presence of both M1 and M2 macrophages, which was reduced with quercetin treatment (**Figures 7I, J**). Furthermore, immunohistochemistry staining for inflammatory mediators, including TNF-α, IL-1β, IL-6, MCP–1, TGF-β and IL-10, suggested that although burn/tenotomy led to increased inflammatory responses, quercetin lowered the accumulation of these cytokines in a dose-dependent manner (**Figures 8A–F**).

### SIRT1 Activation Caused the Modulatory Effect of Quercetin on Immune Responses and Trauma-Induced Heterotopic Ossification

**Figure 9:**
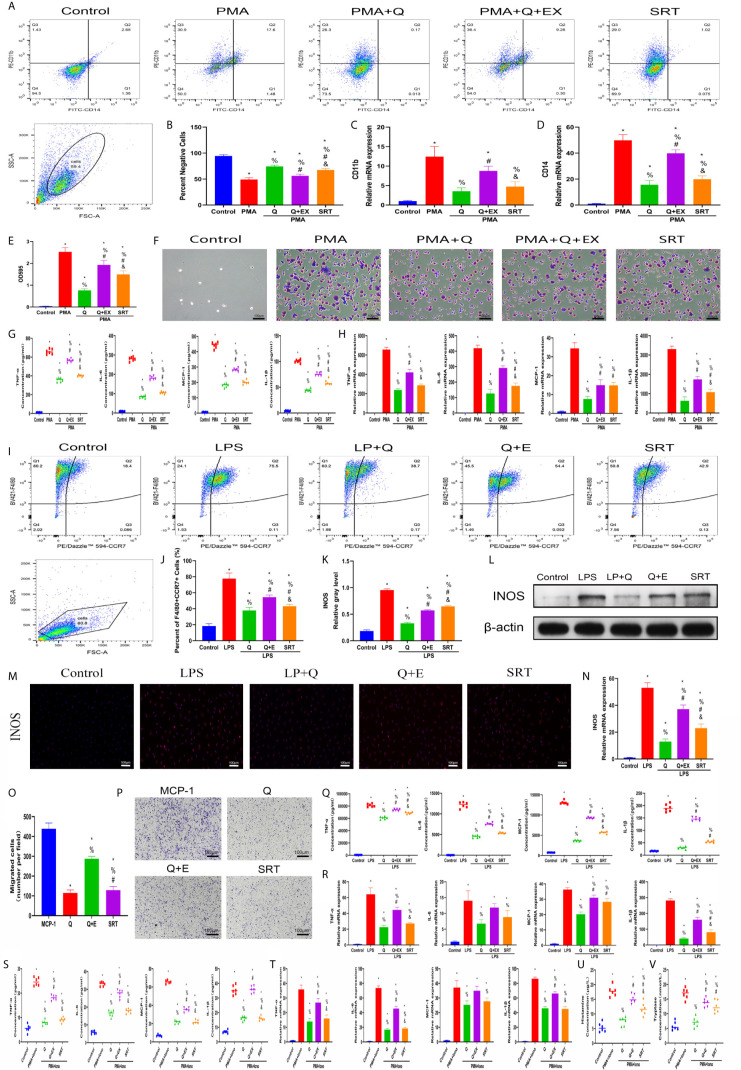
SIRT1 activation was required for the beneficial effects of quercetin on monocyte/macrophage and mast cell behavior. THP-1 cells were pretreated with 20 μM quercetin for 1 h, 10 μM EX527 and 1 μM SRT1720 HCI for 6 h and then incubated with PMA for 48 h or 24 h. **(A)** Flow cytometry analysis for CD14 and CD11b. **(B)** Bar graphs illustrating the percentage of CD14 and CD11b double-negative cells. **(C, D)** The relative gene expression of CD11b and CD14 was checked using qRT-PCR. **(E, F)** Crystal violet staining of the transitioned macrophages from THP-1 and OD595 values of eluted staining. **(G)** Inflammatory cytokine secretion during monocyte-to-macrophage transition. **(H)** The relative gene expression of inflammatory cytokines as tested by qRT-PCR. BMDMs were pretreated with 20 μM quercetin for 1 h, 10 μM EX527 and 1 μM SRT1720 HCI for 6 h and then incubated with LPS for 24 h or 8 h. **(I)** Flow cytometry analysis for F4/80 and CCR7. **(J)** Bar graph illustrating the percentage of F4/80 and CCR7 double-positive cells. **(K, L)** The protein expression of INOS was detected by western blot. **(M)** Immunofluorescence staining of INOS. **(N)** The relative gene expression of INOS was checked using qRT-PCR. **(O, P)** Crystal violet staining of migrated BMDMs after the Transwell assay. **(Q)** Inflammatory cytokines in the supernatants of BMDMs. **(R)** The relative gene expression of inflammatory cytokines. P815 cells were pretreated with 20 μM quercetin for 1 h, 10 μM EX527 and 1 μM SRT1720 HCI for 6 h and then incubated with PMA and ionomycin for 24 h or 8 h. **(S)** Inflammatory cytokines secretion in P815 cells. **(T)** Relative gene expression of inflammatory cytokines in P815 cells. **(U)** Histamine release from the P815 cells. **(V)** Tryptase release from the P815 cells. *P < 0.05 compared with the control group, ^%^P < 0.05 compared with the LPS, PMA, or PMA+Iono group, ^#^P < 0.05 compared with the Q group, ^&^P < 0.05 compared with the Q+EX group.

To confirm that the SIRT1-dependent mechanism was responsible for the beneficial effects of quercetin on inflammatory responses, we further performed rescue experiments *in vitro*. For THP-1 cells, EX527 successfully blocked the inhibitory effects of quercetin on monocyte-to-macrophage transition and concomitant inflammatory cytokine production, whereas SRT1720 HCI partly reproduced the effects of quercetin (**Figures 9A–H**). In BMDMs, EX527 prominently restored cell migration, M1 polarization and cytokine production damage by quercetin. Conversely, SRT1720 HCI significantly inhibited cell migration, M1 polarization and cytokine production, although the effect was somewhat weaker than that of quercetin (**Figures 9I–R**). For P815 cells, the reduced inflammatory cytokine gene expression and secretion by quercetin was rescued by EX527 but mimicked by SRT1720 HCI, which was also observed for histamine and tryptase release (**Figures 9S–V**). Therefore, we suggest that SIRT1 activation is required for the beneficial effects of quercetin.

**Figure 10:**
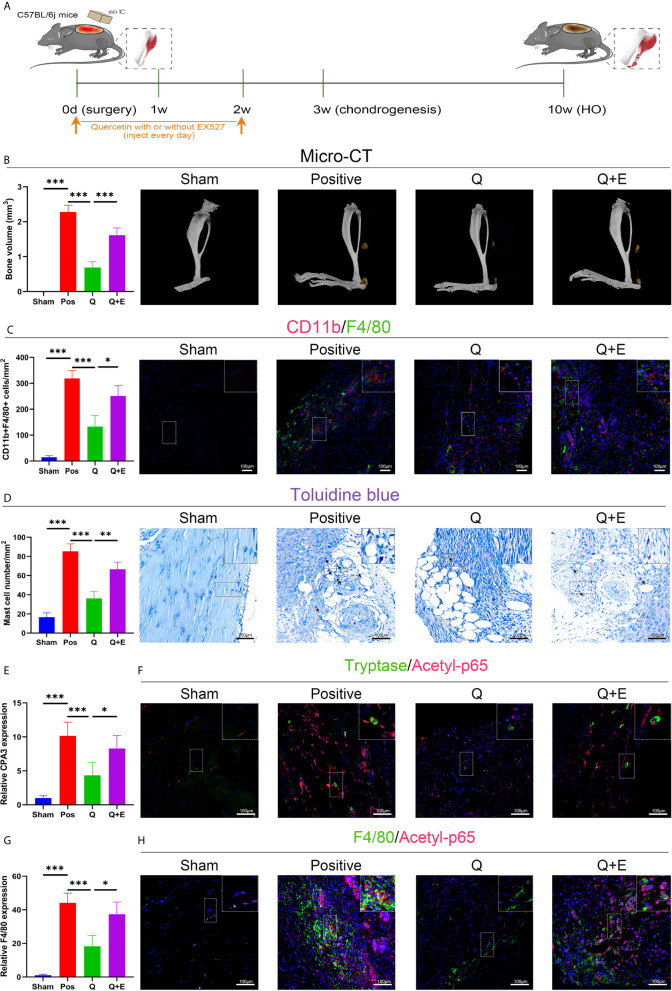
Quercetin impeded immune cell infiltration and HO by activating SIRT1 *in vivo*. **(A)** Schematic depiction of the indicated treatment protocols for animal experiments. **(B)** Micro-CT scanning for observation and quantification of HO formation. **(C)** Double fluorescence staining of CD11b and F4/80 7 days after burn/tenotomy and quantification of CD11b+F4/80+ cells. **(D)** Toluidine blue staining for observation and quantification of mast cells 7 days after burn/tenotomy. **(E)** Relative gene expression of the mast cell marker CPA3 as determined by qRT-PCR. **(F)** Double fluorescence staining of tryptase and acetyl-p65 7 days after burn/tenotomy. **(G)** Relative gene expression of macrophage marker F4/80 as determined by qRT-PCR. **(H)** Double fluorescence staining of F4/80 and acetyl-p65 7 days after burn/tenotomy. Original magnification is 40x. Inserts are approximately 3.5x magnified images of the boxed area. N=4/group. ***P < 0.001.

**Figure 11:**
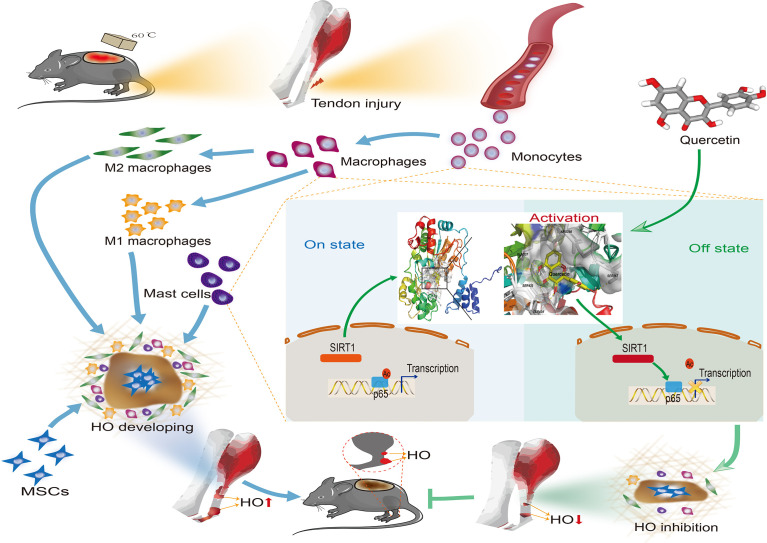
Graphical summary of the effects and mechanisms of quercetin on immune responses that regulate HO progression. Upon tendon injury, monocytes are motivated by circulation and accumulate at the injury site where they undergo monocyte-to-macrophage transition and then are further polarized into the M1 and M2 phenotypes. In parallel, mast cells also respond to injury and infiltrate the injured tissue, synergizing with macrophages to constitute the osteogenic immune microenvironment, within which mesenchymal stem cells (MSCs) are recruited and induced osteochondral differentiation. However, quercetin suppresses the aforementioned monocyte-to-macrophage transition, macrophage polarization and mast cell activation partly by modulating SIRT1/NFκB signaling involved in these biological processes, thereby preventing HO formation.

Accordingly, the SIRT1-dependent mechanism was later confirmed again in the murine burn/tenotomy model (**Figure 10A**). Micro-CT analysis confirmed that in the presence of EX527, quercetin partly lost its inhibitory effect on HO formation (**Figure 10B**). Double fluorescence staining of the monocyte markers CD11b and F4/80 was performed to assess the monocyte-to-macrophage transition *in vivo*. The results showed that both colocalization of CD11b and F4/80 and the staining density of F4/80 were decreased by quercetin, indicating reduced monocyte-derived macrophage infiltration, which was enhanced by EX527 (**Figure 10C**). Toluidine blue staining of mast cells also displayed a trend similar to that of F4/80 and CD11b fluorescence staining (**Figure 10D**). Moreover, qRT-PCR results showed decreased expression of both F4/80 and CPA3 after quercetin, which was rescued by EX527 (**Figures 10E, G**), suggesting that inhibition of SIRT1 restored the diminished mast cell infiltration. Moreover, elevated expression of acetylated NFκB p65 was found in macrophages and mast cells upon burn/tenotomy, which was inhibited by quercetin but reproduced by EX527, revealing that NFκB signaling was implicated in quercetin-mediated immune response modulation and was SIRT1 dependent (**Figures 10F, H**). Taken together, we can infer that quercetin may prevent trauma-induced inflammatory responses and HO by regulating SIRT1/NFκB signaling (**Figure 11**).

## Discussion

Numerous studies have shown that tissue damage at the onset of many diseases could lead to innate immune homeostasis disruption and immune cell recruitment (40, 41). Similarly, for HO, it was also emphasized that immune system activation and accompanying inflammation are present after soft tissue injury, which initiates a permissive niche facilitating HO anlagen formation, making immune cells an appealing target for HO prevention (34, 35, 42). As the predetermination of mesenchymal progenitor cell fate can occur as early as 3 days after injury, the initial inflammatory stage featuring intense immune cell responses is also considered to be the crucial therapeutic time window for HO (43). In line with this evidence, in this study, we confirmed the highly stimulated monocyte/macrophage and mast cell responses after tendon injury and revealed that inhibition of monocyte/macrophage and mast cell infiltration and ensuing proinflammatory activity by quercetin at an early stage effectively inhibited aberrant HO formation.

Macrophages play a fundamental role in the control and remodeling of tissue homeostasis and are primary cellular responders upon injury (35, 44). In addition to the tissue in which resident macrophages originally exist preceding injury, macrophages at the injury site also arise from the circulating pool of monocytes that receive stress signals and differentiate into inflammatory macrophages, which finally outnumber the resident macrophages and become the main initiator of inflammatory reactions (41). In this study, we used F4/80 to mark macrophages and found that macrophages invaded the injured tendon early, from 3 days to 3 weeks, indicating the potential significance of macrophages in early stages of HO. When we performed macrophage depletion using clodronate liposomes, chondrogenesis and subsequent HO were dramatically reduced, consistent with previous reports that accentuated the central role of macrophages in HO (34, 35, 43).

Several studies have also proposed the prevalence of mast cells following tendon injury (45, 46). Accordingly, we found mast cells, as revealed by tryptase and CPA3, present after mouse tenotomy, peaking at 7 days postinjury. Furthermore, toluidine blue staining suggested that tendon injury intensified mast cell degranulation. Mast cells have long been famous for their critical role in mediating allergic reactions. Nonetheless, accumulating data also highlight that mast cells can also act as primary inflammation producers and contribute to soft tissue healing and remodeling. Utilizing cromolyn sodium as a mast cell stabilizer, we also observed decreased HO volume after burn/tenotomy, similar to macrophage depletion. Likewise, Salisbury et al. profoundly reduced HO using cromolyn sodium in a BMP2 delivery model (47). Their discoveries further support an emerging notion that neuroinflammation induces mast cell degranulation as a central mechanism in HO formation (47, 48).

To better make use of this evidence to provide therapeutic modalities for HO, we further explored the underlying molecular mechanism mediating monocyte/macrophage and mast cell recruitment and activation in HO. SIRT1 is a protein deacetylase implicated in posttranslational modification and has been found to modulate multiple biological processes, including immunity (49, 50). Targeting SIRT1 has recently been the focus of researchers because activation of SIRT1 benefits lifespan and combats senescence, especially with natural products (18, 51, 52). Given this evidence, we hypothesized that SIRT1 might be responsible for the dysregulated immune responses at early stages of HO. Consistent with our hypothesis, after burn/tenotomy, we found that SIRT1 expression was prominently restricted. When a SIRT1 agonist was applied, both the mast cells indicated by toluidine blue staining and the CD11b+F4/80+ cells indicated by fluorescence staining were decreased, which finally led to HO inhibition. Collectively, the above findings suggest that SIRT1 is a critical molecular target for immune responses controlling HO and that activation of SIRT1 might exert beneficial effects on HO blockade.

There is a growing list of SIRT1 substrates, including transcription factors for FOXO, p53, and PGC-1. Among them, the NFκB p65 protein is a classical proinflammatory transcription factor responsible for immune response regulation. Acetylation of NFκB p65 at the K310 residue is imperative for full transcriptional activity of NF-κB and activation of canonical NFκB signaling (53). To assess whether deacetylation of NFκB p65 is the downstream event mediating the anti-inflammatory effect of SIRT1, we assayed for the acetylation of NFκB p65 in macrophages and mast cells in a trauma-induced HO model after SRT1720 HCI treatment. Immunostaining of acetylated NFκB p65 was dramatically enhanced upon burn/tenotomy. However, after SRT1720 HCI treatment, acetylated NFκB p65 expression was largely weakened, paralleled by the reduced macrophage and mast cell presence and consistent with the assumption that the immunomodulatory effects of SIRT1 may be due to altered NFκB signaling. In accordance with this finding, prolonged NFκB pathway activation was found in monocytes and macrophages from FOP patients (16), and pharmacological inhibition of canonical NFκB signaling also abrogated HO in a rat brain-traumatic/burn/tenotomy model (54). Altogether, we showed that impaired SIRT1/NFκB signaling was responsible for aberrant immunity during HO development.

Further, to implement immune modulation and SIRT1 targeting effects, we next explored whether quercetin, a natural dietary polyphenol compound described to have immunomodulatory ability, could reduce immune responses after tendon injury and thus decrease HO formation. In this study, the strong binding affinity of quercetin for murine SIRT1 and the potent SIRT1/NFκB signaling regulatory capacity of quercetin were also confirmed, showing a potential opportunity for quercetin to treat HO. Since the intake of dietary polyphenols such as quercetin is correlated with a lower risk of coronary disease and mental disorders and most studies performed on humans rarely reported side effects when taking quercetin as a supplement within 12 weeks (55–57), quercetin also possesses great clinical translational potential. Regarding this, a recent clinical trial has already implicated quercetin as a senolytic drug, which reduced the senescent cell burden in humans (58).

As expected, we discovered that quercetin prevented macrophage recruitment in a dose-dependent manner and significantly abolished HO formation in the burn/tenotomy model. To determine whether the weakened macrophage recruitment was attributed to the abated ability for monocytes to become macrophages, the effect of quercetin on the monocyte-to-macrophage transition was assessed *in vitro*. The results showed that quercetin decreased the PMA-induced monocyte-to-macrophage transition in a dose-dependent manner. Additionally, quercetin also directly suppressed MCP1-induced macrophage migration, which explains the sharply decreased macrophage presence after quercetin treatment *in vivo*. These findings suggest a dual inhibitory effect of quercetin on possible mechanisms of macrophage participation throughout HO progression.

Because macrophages are highly responsive and programmable, they can polarize under distinct environmental stimuli to show differential behavior and regulate tissue homeostasis. Generally, according to a widely accepted classification based on specific cell markers, the macrophage phenotype can be divided into classically activated and alternatively activated phenotypes, abbreviated as M1 and M2, respectively. It is well documented in the literature that M1 and M2 synergistically participate in the healing response after musculoskeletal injury. Considering HO to be a result of aberrant healing, we also found that both M1 and M2 infiltrated the regenerated tissue at the early stage after tendon injury, similar to what was observed during the repair process of muscle injury and bone fracture (59). However, quercetin prominently inhibited macrophage polarization to either the M1 or M2 subtype both *in vitro* and *in vivo*. A previous study demonstrated that depletion of M1 during early bone repair led to decreased soft tissue calcification and negatively affected bone mineralization (60). Other studies have already suggested the excellent promoting effects of M2 on the commitment of stem cells to the osteoblastic lineage (61, 62). Thus, the reduced M1 and M2 infiltration is supposed to be detrimental for the endochondral ossification process during HO progression. Interestingly, single-cell sequencing performed by Sorkin et al. (43) indicated that the M1 markers IL-1β and TNF-α can colocalize with the M2 markers TGF-β1 or Arg1 to define the monocyte/macrophage clusters that appear after tendon injury, suggesting that a higher level of heterogeneity of monocyte/macrophage clusters might be involved and beyond the traditional dichotomy of the M1 and M2 subtypes. In this regard, the weakened M1 and M2 marker expression at the injury site might also be cautiously regarded as a reflection of the impaired macrophage response. Given the evidence (44) that immune responses are overactive at early stages and application of immunosuppressants at early stages significantly limits HO development, the dampened initial overactivation of macrophages by quercetin might also constitute an osteogenic-suppressive immune microenvironment that inhibits aberrant HO formation, which was indicated by diminished PDGFRα+ mesenchymal stem cell accumulation. As previous study also indicated that quercetin could dose-dependently promote osteogenic differentiation of stem cells (63–65), the inhibitory effects of quercetin on HO might primarily attribute to its immunomodulatory property.

During activation, mast cells can release potent cytokines and enzymes, including MCP-1, TNF-α, and tryptase, interacting with surrounding cells and finally triggering tissue remodeling (17). However, in this study, our findings suggest that quercetin also decreased the mast cell number and activation to some extent, which possibly contributed to HO blockade. Taken together, it could be speculated that quercetin attenuated trauma-induced HO at least partly through combined monocyte/macrophage and mast cell influx restraint. Similar to these results, the combined abolishment of mast cell and macrophage activity also demonstrated joint efforts in a mouse model of fibrodysplasia ossificans progressive (FOP) (12), a genetic form of HO.

One of the principal proinflammatory activities of immune cells favoring parenchymal proliferation and differentiation is the secretion of various inflammatory cytokines. The link between these proinflammatory cytokines and osteogenesis has already been recognized by researchers. TNF-α and IL-6 can promote ossification of primary ligament cells from patients, and TNF-α priming or IL-6 intervention endows mesenchymal cells with a greater potential for osteogenesis (66). TGF-β is a long-established pleiotropic contributor to HO and can be activated by proteases released during mast cell activation (34, 67). Moreover, IL-1β can synergize with TGF-β to induce mesenchymal phenotype acquisition in endothelial cells and endow them with osteogenic lineage commitment potential (68), a phenomenon called the endothelial to mesenchymal transition (EndMT), which is commonly seen in tissue healing disorders. In addition, both MCP-1 and IL-10 have been identified as predictive biomarkers for HO occurrence after blast injury (15, 69), and IL-10 has recently been identified to be the key factor for macrophage-promoted osteogenesis of stem cells (62). Therefore, the inhibitory effect of quercetin on inflammatory factor synthesis and secretion was evaluated in this study. During monocyte-to-macrophage transition, macrophage polarization and mast cell activation *in vitro*, quercetin significantly diminished TNF-α, IL-1β, IL-6, MCP–1, TGF-β and IL-10 release in a dose-dependent manner. In regard to animal models, these inflammatory factors induced by burn/tenotomy injury were effectively lowered by quercetin. The anti-inflammatory effect of quercetin has also been revealed in other studies. Hu et al. reported that quercetin protected the brain from neuroinflammation in db/db mice (70). Studies implemented by Ding et al. also found that quercetin abated mast cell degranulation in allergic conjunctivitis (71). However, to specify the roles of the above proinflammatory cytokines and confirm their necessity in trauma-induced HO, additional investigations incorporating specific knockout models are warranted.

Nonetheless, there are some limitations in our study. First, although there is no consensus about the gold-standard model of trauma-induced HO and although the murine burn/tenotomy model does reflect the classical pathological paradigm, including the inflammatory events and pro-osteogenic local niches in HO formation, the absence of bone trauma and mechanical loading often manifested in actual clinical settings might confound the clinical interpretation and translation of the findings in this study. Therefore, a larger animal with similar anatomy to humans should be studied to determine the pharmacological effects and safety of quercetin. Second, the knockdown or overexpression strategy we used in this study utilized chemical agonists or antagonists. Since the target specificity of these small molecular chemicals cannot be completely confirmed and conclusions drawn from knockout paradigms can vary from those of knockdown paradigms (72), what we found in this study should be confirmed in SIRT1 transgenic cells or mice, which could provide stronger evidence.

## Conclusion

As described above, our results showed that the low-cost and readily available polyphenol quercetin has a previously unrecognized ability to inhibit trauma-induced HO by mitigating inflammatory responses through SIRT1 activation. Moreover, targeting SIRT1/NFκB signaling is beneficial for tuning dysregulated immune responses contributing to HO, which could provide insights into future drug treatments.

## Data Availability Statement

The raw data supporting the conclusions of this article will be made available by the authors, without undue reservation.

## Ethics Statement

The animal study was reviewed and approved by Institutional Animal Care and Use Committee (IACUC) of the Shanghai Sixth People’s Hospital.

## Author Contributions

Conceptualization and funding acquisition: CF. Study design and manuscript writing: YQ, JL, ZS. Experiment conduction: JL, ZS, GL. Data collection, statistical analysis, and formal analysis: GL, SW, ZY, HC, HX, YH. All authors contributed to the article and approved the submitted version.

## Funding

This work was supported by the National Natural Science Foundation of China (81830076, 82002290, 81672146), Shanghai Sailing Program (No. 20YF1436000), Science and Technology Development Fund of Shanghai Pudong New Area (PKJ2018-Y55, PKJ2018-Y52) and a grant from Science Foundation of Shanghai Health and Family Planning Commission (20174Y0225).

## Conflict of Interest

The authors declare that the research was conducted in the absence of any commercial or financial relationships that could be construed as a potential conflict of interest.

## References

[1] Xu R Hu J Zhou X Yang Y. Heterotopic Ossification: Mechanistic Insights and Clinical Challenges. Bone (2018) 109:134–42. 10.1016/j.bone.2017.08.025 28855144

[2] Cholok D Chung MT Ranganathan K Ucer S Day D Davis TA. Heterotopic Ossification and the Elucidation of Pathologic Differentiation. Bone (2018) 109:12–21. 10.1016/j.bone.2017.09.019 28987285 PMC6585944

[3] Potter BK Forsberg JA Davis TA Evans KN Hawksworth JS Tadaki D. Heterotopic Ossification Following Combat-Related Trauma. J Bone Joint Surg Am (2010) 92 Suppl 2:74–89. 10.2106/JBJS.J.00776 21123594

[4] Daniels CM Pavey GJ Arthur J Noller M Forsberg JA Potter BK. Has the Proportion of Combat-Related Amputations That Develop Heterotopic Ossification Increased? J Orthop Trauma (2018) 32(6):283–7. 10.1097/BOT.0000000000001158 29533305

[5] Levi B Jayakumar P Giladi A Jupiter JB Ring DC Kowalske K. Risk Factors for the Development of Heterotopic Ossification in Seriously Burned Adults: A National Institute on Disability, Independent Living and Rehabilitation Research Burn Model System Database Analysis. J Trauma Acute Care Surg (2015) 79(5):870–6. 10.1097/TA.0000000000000838 PMC4621805 26496115

[6] Bargellesi S Cavasin L Scarponi F De Tanti A Bonaiuti D Bartolo M. Occurrence and Predictive Factors of Heterotopic Ossification in Severe Acquired Brain Injured Patients During Rehabilitation Stay: Cross-Sectional Survey. Clin Rehabil (2018) 32(2):255–62. 10.1177/0269215517723161 28805078

[7] Andarawis-Puri N Flatow EL Soslowsky LJ. Tendon Basic Science: Development, Repair, Regeneration, and Healing. J Orthop Res (2015) 33(6):780–4. 10.1002/jor.22869 PMC4427041 25764524

[8] Bachman DR Fitzsimmons JS O’Driscoll SW. Safety of Arthroscopic Versus Open or Combined Heterotopic Ossification Removal Around the Elbow. Arthroscopy (2019) 36(2):422–30. 10.1016/j.arthro.2019.09.010 31870750

[9] Sun Z Cui H Ruan J Li J Wang W Fan C. What Range of Motion and Functional Results Can be Expected After Open Arthrolysis With Hinged External Fixation for Severe Posttraumatic Elbow Stiffness? Clin Orthop Relat Res (2019) 477(10):2319–28. 10.1097/CORR.0000000000000726 PMC6999955 31107330

[10] Kodde IF van Rijn J van den Bekerom MPJ Eygendaal D. Surgical Treatment of Post-Traumatic Elbow Stiffness: A Systematic Review. J Shoulder Elbow Surg (2013) 22(4):574–80. 10.1016/j.jse.2012.11.010 23375881

[11] Lee EK Namdari S Hosalkar HS Keenan MA Baldwin KD. Clinical Results of the Excision of Heterotopic Bone Around the Elbow: A Systematic Review. J Shoulder Elbow Surg (2013) 22(5):716–22. 10.1016/j.jse.2012.11.020 23380078

[12] Convente MR Chakkalakal SA Yang E Caron RJ Zhang D Kambayashi T. Depletion of Mast Cells and Macrophages Impairs Heterotopic Ossification in an Acvr1(R206H) Mouse Model of Fibrodysplasia Ossificans Progressiva. J Bone Miner Res (2018) 33(2):269–82. 10.1002/jbmr.3304 PMC7737844 28986986

[13] Wang C Song W Chen B Liu X He Y. Exosomes Isolated From Adipose-Derived Stem Cells: A New Cell-Free Approach to Prevent the Muscle Degeneration Associated With Torn Rotator Cuffs. Am J Sports Med (2019) 47(13):3247–55. 10.1177/0363546519876323 31560856

[14] Matsuo K Chavez RD Barruet E Hsiao EC. Inflammation in Fibrodysplasia Ossificans Progressiva and Other Forms of Heterotopic Ossification. Curr Osteoporos Rep (2019) 17(6):387–94. 10.1007/s11914-019-00541-x PMC7271746 31721068

[15] Sung Hsieh HH Chung MT Allen RM Ranganathan K Habbouche J Cholok D. Evaluation of Salivary Cytokines for Diagnosis of Both Trauma-Induced and Genetic Heterotopic Ossification. Front Endocrinol (Lausanne) (2017) 8:74. 10.3389/fendo.2017.00074 28484423 PMC5401868

[16] Barruet E Morales BM Cain CJ Ton AN Wentworth KL Chan TV. NF-κB/MAPK Activation Underlies ACVR1-mediated Inflammation in Human Heterotopic Ossification. JCI Insight (2018) 3(22):e122958. 10.1172/jci.insight.122958 PMC6302947 30429363

[17] Mukai K Tsai M Saito H Galli SJ. Mast Cells as Sources of Cytokines, Chemokines, and Growth Factors. Immunol Rev (2018) 282(1):121–50. 10.1111/imr.12634 PMC5813811 29431212

[18] Dehghan E Goodarzi M Saremi B Lin R Mirzaei H. Hydralazine Targets cAMP-dependent Protein Kinase Leading to sirtuin1/5 Activation and Lifespan Extension in C. elegans. Nat Commun (2019) 10(1):4905. 10.1038/s41467-019-12425-w PMC6817882 31659167

[19] Ma R Wu Y Zhai Y Hu B Ma W Yang W. Exogenous Pyruvate Represses Histone Gene Expression and Inhibits Cancer Cell Proliferation Via the NAMPT-NAD+-SIRT1 Pathway. Nucleic Acids Res (2019) 47(21):11132–50. 10.1093/nar/gkz864 PMC6868375 31598701

[20] Cao R Wang G Qian K Chen L Ju L Qian G. TM4SF1 Regulates Apoptosis, Cell Cycle and ROS Metabolism Via the PPARγ-SIRT1 Feedback Loop in Human Bladder Cancer Cells. Cancer Lett (2018) 414:278–93. 10.1016/j.canlet.2017.11.015 29175458

[21] Nakamura K Kageyama S Ke B Fujii T Sosa RA Reed EF. Sirtuin 1 Attenuates Inflammation and Hepatocellular Damage in Liver Transplant Ischemia/Reperfusion: From Mouse to Human. Liver Transpl (2017) 23(10):1282–93. 10.1002/lt.24821 PMC5705033 28719070

[22] Liu L Zhou M Zhu R Zhou J Ni L Wang Z. Hydrogen Sulfide Protects Against Particle-Induced Inflammatory Response and Osteolysis Via SIRT1 Pathway in Prosthesis Loosening. FASEB J (2020) 34(3):3743–54. 10.1096/fj.201900393RR 31943384

[23] Limagne E Thibaudin M Euvrard R Berger H Chalons P Végan F. Sirtuin-1 Activation Controls Tumor Growth by Impeding Th17 Differentiation Via STAT3 Deacetylation. Cell Rep (2017) 19(4):746–59. 10.1016/j.celrep.2017.04.004 28445726

[24] Joice M Vasileiadis GI Amanatullah DF. Non-Steroidal Anti-Inflammatory Drugs for Heterotopic Ossification Prophylaxis After Total Hip Arthroplasty: A Systematic Review and Meta-Analysis. Bone Joint J (2018) 100-B(7):915–22. 10.1302/0301-620X.100B7.BJJ-2017-1467.R1 29954195

[25] Dogné J-M Hanson J Supuran C Pratico D. Coxibs and Cardiovascular Side-Effects: From Light to Shadow. Curr Pharm Des (2006) 12(8):971–5. 10.2174/138161206776055949 16533164

[26] Solomon DH Shao M Wolski K Nissen S Husni ME Paynter N. Derivation and Validation of a Major Toxicity Risk Score Among Nonsteroidal Antiinflammatory Drug Users Based on Data From a Randomized Controlled Trial. Arthritis Rheumatol (2019) 71(8):1225–31. 10.1002/art.40870 30801994

[27] Galleggiante V De Santis S Liso M Verna G Sommella E Mastronardi M. Quercetin-Induced miR-369-3p Suppresses Chronic Inflammatory Response Targeting C/EBP-β. Mol Nutr Food Res (2019) 63(19):e1801390–e. 10.1002/mnfr.201801390 31338984

[28] Tan R-Z Wang C Deng C Zhong X Yan Y Luo Y. Quercetin Protects Against Cisplatin-Induced Acute Kidney Injury by Inhibiting Mincle/Syk/NF-κB Signaling Maintained Macrophage Inflammation. Phytother Res (2020) 34(1):139–52. 10.1002/ptr.6507 31497913

[29] Saccol R da Silveira KL Manzoni AG Abdalla FH de Oliveira JS Dornelles GL. Antioxidant, Hepatoprotective, Genoprotective, and Cytoprotective Effects of Quercetin in a Murine Model of Arthritis. J Cell Biochem (2019) 121(4):2792–801. 10.1002/jcb.29502 31691375

[30] Sun Z Zeng J Wang W Jia X Wu Q Yu D. Magnoflorine Suppresses MAPK and NF-κB Signaling to Prevent Inflammatory Osteolysis Induced by Titanium Particles In Vivo and Osteoclastogenesis Via RANKL In Vitro. Front Pharmacol (2020) 11:389. 10.3389/fphar.2020.00389 32300300 PMC7142243

[31] Huang J Lin D Wei Z Li Q Zheng J Zheng Q. Parathyroid Hormone Derivative With Reduced Osteoclastic Activity Promoted Bone Regeneration Via Synergistic Bone Remodeling and Angiogenesis. Small (2020) 16(6):e1905876. 10.1002/smll.201905876 31962381

[32] Peterson JR Agarwal S Brownley RC Loder SJ Ranganathan K Cederna PS. Direct Mouse Trauma/Burn Model of Heterotopic Ossification. J Vis Exp (2015) 102):e52880–e. 10.3791/52880 PMC4544908 26274052

[33] Zhang J Wang L Cao H Chen N Yan B Ao X. Neurotrophin-3 Acts on the Endothelial-Mesenchymal Transition of Heterotopic Ossification in Rats. J Cell Mol Med (2019) 23(4):2595–609. 10.1111/jcmm.14150 PMC6433730 30672120

[34] Wang X Li F Xie L Crane J Zhen G Mishina Y. Inhibition of Overactive TGF-β Attenuates Progression of Heterotopic Ossification in Mice. Nat Commun (2018) 9(1):551–. 10.1038/s41467-018-02988-5 PMC5803194 29416028

[35] Zhang J Wang L Chu J Ao X Jiang T Bin Y. Macrophage-Derived Neurotrophin-3 Promotes Heterotopic Ossification in Rats. Lab Invest (2020) 100(5):762–76. 10.1038/s41374-019-0367-x 31896816

[36] Torossian F Guerton B Anginot A Alexander KA Desterke C Soave S. Macrophage-Derived Oncostatin M Contributes to Human and Mouse Neurogenic Heterotopic Ossifications. JCI Insight (2017) 2(21):1–12. 10.1172/jci.insight.96034 PMC5752299 29093266

[37] Salisbury EA Lazard ZW Ubogu EE Davis AR Olmsted-Davis EA. Transient Brown Adipocyte-Like Cells Derive From Peripheral Nerve Progenitors in Response to Bone Morphogenetic Protein 2. Stem Cells Transl Med (2012) 1(12):874–85. 10.5966/sctm.2012-0090 PMC3659676 23283549

[38] Kan L Mutso AA McGuire TL Apkarian AV Kessler JA. Opioid Signaling in Mast Cells Regulates Injury Responses Associated With Heterotopic Ossification. Inflammation Res (2014) 63(3):207–15. 10.1007/s00011-013-0690-4 PMC4887745 24327087

[39] Lees-Shepard JB Goldhamer DJ. Stem Cells and Heterotopic Ossification: Lessons From Animal Models. Bone (2018) 109:178–86. 10.1016/j.bone.2018.01.029 PMC5866227 29409971

[40] Dakin SG Martinez FO Yapp C Wells G Oppermann U Dean BJF. Inflammation Activation and Resolution in Human Tendon Disease. Sci Transl Med (2015) 7(311):311ra173–311ra173. 10.1126/scitranslmed.aac4269 PMC4883654 26511510

[41] Arnold L Henry A Poron F Baba-Amer Y van Rooijen N Plonquet A. Inflammatory Monocytes Recruited After Skeletal Muscle Injury Switch Into Antiinflammatory Macrophages to Support Myogenesis. J Exp Med (2007) 204(5):1057–69. 10.1084/jem.20070075 PMC2118577 17485518

[42] Li L Jiang Y Lin H Shen H Sohn J Alexander PG. Muscle Injury Promotes Heterotopic Ossification by Stimulating Local Bone Morphogenetic Protein-7 Production. J Orthop Translat (2019) 18:142–53. 10.1016/j.jot.2019.06.001 PMC6718974 31508317

[43] Sorkin M Huber AK Hwang C WFt C Menon R Li J. Regulation of Heterotopic Ossification By Monocytes in a Mouse Model of Aberrant Wound Healing. Nat Commun (2020) 11(1):722–. 10.1038/s41467-019-14172-4 PMC7002453 32024825

[44] Kan C Yang J Na D Xu Y Yang B Zhao H. Inhibition of Immune Checkpoints Prevents Injury-Induced Heterotopic Ossification. Bone Res (2019) 7:33–. 10.1038/s41413-019-0074-7 PMC6823457 31700694

[45] Alim MA Ackermann PW Eliasson P Blomgran P Kristiansson P Pejler G. Increased Mast Cell Degranulation and Co-Localization of Mast Cells With the NMDA Receptor-1 During Healing After Achilles Tendon Rupture. Cell Tissue Res (2017) 370(3):451–60. 10.1007/s00441-017-2684-y 28975451

[46] Zhang X Yao H Qian Q Li N Jin W Qian Y. Cerebral Mast Cells Participate in Postoperative Cognitive Dysfunction by Promoting Astrocyte Activation. Cell Physiol Biochem (2016) 40(1-2):104–16. 10.1159/000452528 27855371

[47] Salisbury E Rodenberg E Sonnet C Hipp J Gannon FH Vadakkan TJ. Sensory Nerve Induced Inflammation Contributes to Heterotopic Ossification. J Cell Biochem (2011) 112(10):2748–58. 10.1002/jcb.23225 PMC3329372 21678472

[48] Kan L Lounev VY Pignolo RJ Duan L Liu Y Stock SR. Substance P Signaling Mediates BMP-dependent Heterotopic Ossification. J Cell Biochem (2011) 112(10):2759–72. 10.1002/jcb.23259 PMC3508732 21748788

[49] Hwang JW Yao H Caito S Sundar IK Rahman I. Redox Regulation of SIRT1 in Inflammation and Cellular Senescence. Free Radic Biol Med (2013) 61:95–110. 10.1016/j.freeradbiomed.2013.03.015 23542362 PMC3762912

[50] Yu Q Dong L Li Y Liu G. SIRT1 and HIF1α Signaling in Metabolism and Immune Responses. Cancer Lett (2018) 418:20–6. 10.1016/j.canlet.2017.12.035 29306019

[51] Cătană C-S Atanasov AG Berindan-Neagoe I. Natural Products With Anti-Aging Potential: Affected Targets and Molecular Mechanisms. Biotechnol Adv (2018) 36(6):1649–56. 10.1016/j.biotechadv.2018.03.012 29597027

[52] Igarashi M Guarente L. mTORC1 and SIRT1 Cooperate to Foster Expansion of Gut Adult Stem Cells During Calorie Restriction. Cell (2016) 166(2):436–50. 10.1016/j.cell.2016.05.044 27345368

[53] Chen L-F Mu Y Greene WC. Acetylation of RelA at Discrete Sites Regulates Distinct Nuclear Functions of NF-kappaB. EMBO J (2002) 21(23):6539–48. 10.1093/emboj/cdf660 PMC136963 12456660

[54] Ju J Yu D Xue F Zhao Y Shi W Pan M. Inhibition of NF-κB Prevents Trauma-Induced Heterotopic Ossification in Rat Model. Connect Tissue Res (2019) 60(3):304–10. 10.1080/03008207.2018.1530771 30288996

[55] Gollucke APB Peres RC Odair A Jr. Ribeiro DA. Polyphenols: A Nutraceutical Approach Against Diseases. Recent Pat Food Nutr Agric (2013) 5(3):214–9. 10.2174/2212798405666131129153239 24294942

[56] Yamagata K. Polyphenols Regulate Endothelial Functions and Reduce the Risk of Cardiovascular Disease. Curr Pharm Des (2019) 25(22):2443–58. 10.2174/1381612825666190722100504 31333108

[57] Andres S Pevny S Ziegenhagen R Bakhiya N Schäfer B Hirsch-Ernst KI. Safety Aspects of the Use of Quercetin as a Dietary Supplement. Mol Nutr Food Res (2018) 62(1):1–63. 10.1002/mnfr.201700447 29127724

[58] Hickson LJ Langhi Prata LGP Bobart SA Evans TK Giorgadze N Hashmi SK. Senolytics Decrease Senescent Cells in Humans: Preliminary Report From a Clinical Trial of Dasatinib Plus Quercetin in Individuals With Diabetic Kidney Disease. EBioMedicine (2019) 47:446–56. 10.1016/j.ebiom.2019.08.069 PMC6796530 31542391

[59] McCauley J Bitsaktsis C Cottrell J. Macrophage Subtype and Cytokine Expression Characterization During the Acute Inflammatory Phase of Mouse Bone Fracture Repair. J Orthop Res (2020) 38(8):1693–702. 10.1002/jor.24603 31989683

[60] Hozain S Cottrell J. Cdllb+ Targeted Depletion of Macrophages Negatively Affects Bone Fracture Healing. Bone (2020) 138:115479. 10.1016/j.bone.2020.115479 32535290

[61] Schlundt C El Khassawna T Serra A Dienelt A Wendler S Schell H. Macrophages in Bone Fracture Healing: Their Essential Role in Endochondral Ossification. Bone (2018) 106:78–89. 10.1016/j.bone.2015.10.019 26529389

[62] Mahon OR Browe DC Gonzalez-Fernandez T Pitacco P Whelan IT Von Euw S. Nano-Particle Mediated M2 Macrophage Polarization Enhances Bone Formation and MSC Osteogenesis in an IL-10 Dependent Manner. Biomaterials (2020) 239:119833. 10.1016/j.biomaterials.2020.119833 32062479

[63] Wang N Wang L Yang J Wang Z Cheng L. Quercetin Promotes Osteogenic Differentiation and Antioxidant Responses of Mouse Bone Mesenchymal Stem Cells Through Activation of the AMPK/SIRT1 Signaling Pathway. Phytother Res (2021) 1–12. 10.1002/ptr.7010 33421256

[64] Zhang Q Chang B Zheng G Du S Li X. Quercetin Stimulates Osteogenic Differentiation of Bone Marrow Stromal Cells Through miRNA-206/connexin 43 Pathway. Am J Transl Res (2020) 12(5):2062–70. PMC7270039 32509200

[65] Kim YJ Bae YC Suh KT Jung JS. Quercetin, a Flavonoid, Inhibits Proliferation and Increases Osteogenic Differentiation in Human Adipose Stromal Cells. Biochem Pharmacol (2006) 72(10):1268–78. 10.1016/j.bcp.2006.08.021 16996034

[66] Noronha N Mizukami A Caliári-Oliveira C Cominal JG Rocha JLM Covas DT. Priming Approaches to Improve the Efficacy of Mesenchymal Stromal Cell-Based Therapies. Stem Cell Res Ther (2019) 10(1):131–. 10.1186/s13287-019-1224-y PMC6498654 31046833

[67] Pejler G. The Emerging Role of Mast Cell Proteases in Asthma. Eur Respir J (2019) 54(4):1900685. 10.1183/13993003.00685-2019 31371445

[68] Maleszewska M Moonen J-RAJ Huijkman N van de Sluis B Krenning G Harmsen MC. IL-1β and TGFβ2 Synergistically Induce Endothelial to Mesenchymal Transition in an NFκB-Dependent Manner. Immunobiology (2013) 218(4):443–54. 10.1016/j.imbio.2012.05.026 22739237

[69] Evans KN Forsberg JA Potter BK Hawksworth JS Brown TS Andersen R. Inflammatory Cytokine and Chemokine Expression is Associated With Heterotopic Ossification in High-Energy Penetrating War Injuries. J Orthop Trauma (2012) 26(11):e204–e13. 10.1097/BOT.0b013e31825d60a5 22588530

[70] Hu T Lu X-Y Shi J-J Liu X-Q Chen Q-B Wang Q. Quercetin Protects Against Diabetic Encephalopathy Via SIRT1/NLRP3 Pathway in db/db Mice. J Cell Mol Med (2020) 24(6):3446–59. 10.1111/jcmm.15026 PMC7131910 32000299

[71] Ding Y Li C Zhang Y Ma P Zhao T Che D. Quercetin as a Lyn Kinase Inhibitor Inhibits IgE-mediated Allergic Conjunctivitis. Food Chem Toxicol (2020) 135:110924–. 10.1016/j.fct.2019.110924 31672514

[72] Wilkinson MF. Genetic Paradox Explained by Nonsense. Nature (2019) 568(7751):179–80. 10.1038/d41586-019-00823-5 PMC10984164 30962551

