# Supplementary material for: Quercetin Attenuates Trauma-Induced Heterotopic Ossification by Tuning Immune Cell Infiltration and Related Inflammatory Insult
Source: Front Immunol. 2021 May 20;12:649285. doi: 10.3389/fimmu.2021.649285 (PMC8173182; doi:10.3389/fimmu.2021.649285)
Supplement: Supplementary file 1 [file DataSheet_1.doc]

**SUPPLEMENTARY INFORMATION**

**
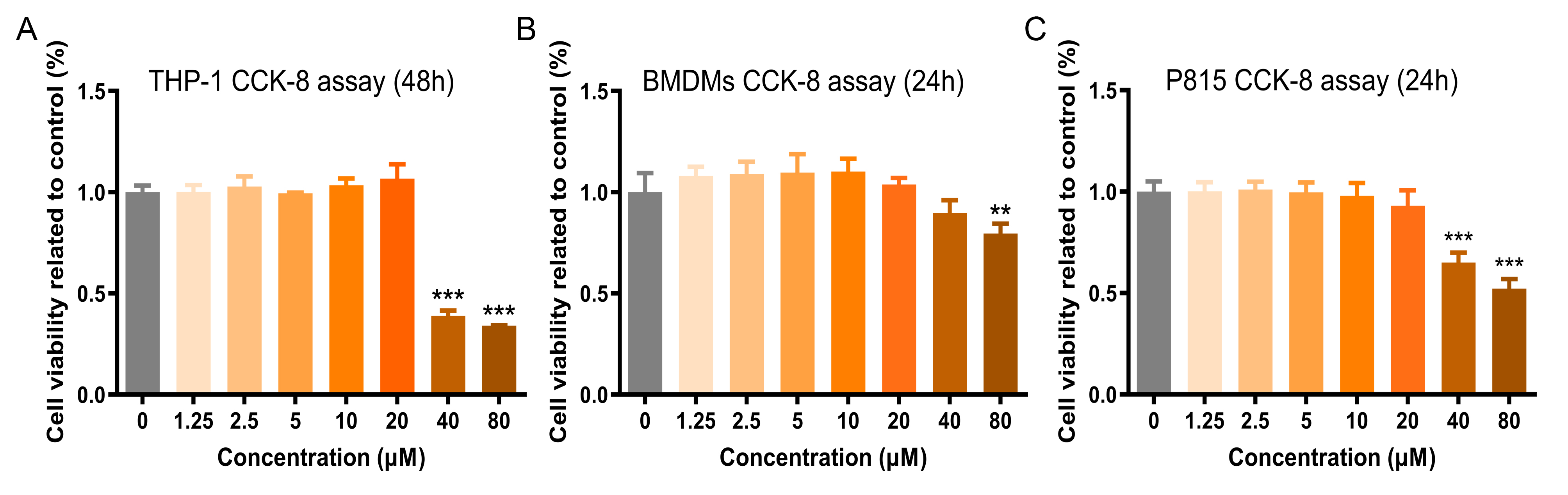
**

**SUPPLEMENTARY FIGURE 1 In vitro cytotoxicity of quercetin on monocytes, macrophages and mast cells.**

THP-1, BMDMs and P815 cells were incubated with different concentrations of quercetin for indicated times. Cell viability of THP-1 (A), BMDMs (B), and P815 (C) related to control group (group without quercetin treatment) was calculated according to CCK8 results. ***P* < 0.01, ****P* < 0.001.

**SUPPLEMENTARY TABLE 1 Real-time PCR primers**

| **Gene** | **Forward** | **Reverse** |
| --- | --- | --- |
| **Murine F4/80** | **CAGTCAGATGATTCAGACGGAGT** | **GGTCACAGTGCCACCAACAA** |
| **Murine CPA3** | **CAAACTGCCTCCTAACCACCA** | **AGCACGTTTCAAAACAAGGGC** |
| **Murine CCR7** | **ACGCAACTTTGAGCGGAACA** | **ACGCCGATGAAGGCATACAA** |
| **Murine Arg1** | **AGGAAAGCTGGTCTGCTGGAA** | **AGATGCTTCCAACTGCCAGAC** |
| **Murine TGF-β1** | **CAAGGGCTACCATGCCAACT** | **GTACTGTGTGTCCAGGCTCCAA** |
| **Murine IL-10** | **AAGGCAGTGGAGCAGGTGAA** | **CCAGCAGACTCAATACACAC** |
| **Murine IL-1β** | **CACTACAGGCTCCGAGATGAAC** | **TCCATCTTCTTCTTTGGGTATTGC** |
| **Murine TNF-α** | **TAGCCAGGAGGGAGA ACAGA** | **CCAGTGAGTGAAAGGGACAGA** |
| **Murine MCP-1** | **CATCCACTACCTTTTCCACAA** | **CATCACAGTCCGAGTCACAC** |
| **Murine IL-6** | **ACCAAGACCATCCAATTCATC** | **CTGACCACAGTGAGGAATGTC** |
| **Murine GAPDH** | **CCTCGTCCCGTAGACAAAATG** | **TGAGGTCAATGAAGGGGTCGT** |
| **Human CD11b** | **TAATACCATCGCATCCAAGCC** | **ACTCCACCAGCCCAGTCATAG** |
| **Human CD14** | **AGAACCTTGTGAGCTGGACGA** | **AGTTCCTTGAGGCGGGAGTAC** |
| **Human IL-1β** | **CGATCACTGAACTGCACGCTC** | **ACAAAGGACATGGAGAACACCACTT** |
| **Human TNF-α** | **CTCTTCTGCCTGCTGCACTTTG** | **ATGGGCTACAGGCTTGTCACTC** |
| **Human MCP-1** | **GAAAGTCTCTGCCGCCCTTC** | **TGCACTGAGATCTTCCTATTGGT** |
| **Human IL-6** | **TACCCCCAGGAGAAGATTCC** | **AGTGCCTCTTTGCTGCTTTC** |
| **Human GAPDH** | **GGAAGCTTGTCATCAATGGAAATC** | **TGATGACCCTTTTGGCTCCC** |
